# Green companions: Affordances of human–tree relationships

**DOI:** 10.1007/s13280-024-02098-1

**Published:** 2025-01-27

**Authors:** Kaisa Kristiina Vainio, Tuomo Takala, Juul Limpens, Karoliina Lummaa, Aino Korrensalo, Aleksi Räsänen, Eeva-Stiina Tuittila

**Affiliations:** 1 School of Forest Sciences, University of Eastern Finland, Joensuu, Finland; 2 University of Turku, Turku, Finland; 3 Department of Environmental Sciences, Wageningen University, Wageningen, The Netherlands; 4 Department of History Culture and Art Studies, University of Turku, Turku, Finland & BIOS Research Unit, Helsinki, Finland; 5 Department of Environmental and Biological Sciences, University of Eastern Finland & Natural Resources Institute Finland, Joensuu, Finland; 6 Natural Resources Institute Finland, Oulu, Finland; 7 Geography Research Unit, University of Oulu, Oulu, Finland

**Keywords:** Affect, Affordance theory, Cultural ecosystem services, Cultural plant studies, Human-nature relationship, Urban planning

## Abstract

Trees offer multiple benefits, including impacts on physical and mental health. In this interdisciplinary study, we explored the relationships humans develop with specific favourite trees based on our survey data (*n* = 158) collected in the Netherlands. Here, we examined action possibilities (affordances) provided by trees, including immaterial actions, such as memorisation or the enjoyment of beauty. We implemented the *affordance theory* with mixed-method analysis. We identified three distinct types of human-arboreal relationships offering a different variety of action possibilities: *nostalgic relationships*, *nurturing relationships,* and *empowering relationships*. We assume that when humans form relationships with trees, the essence of trees is not their characteristics, but the possibilities for action. We recommend that urban green spaces would support interactions between humans and trees. By strengthening our connectedness with nature, it is possible to promote both human well-being and to strengthen environmentally responsible values and behaviours.

**Supplementary Information:**

The online version contains supplementary material available at 10.1007/s13280-024-02098-1.

## Introduction

Why do trees matter? Trees are in many ways central to human wellbeing. Trees offer a wide variety of material and intangible benefits for humans, and create a more pleasant, healthy and comfortable environment to live in. Trees are not only attractive or pleasant to be around, but they also have many ways of affecting the well-being and meaningful life of humans (Jones 2011; Hadavi 2017). The aesthetic, recreational, and other sociocultural values of trees to humans are nowadays widely approached in the context of cultural ecosystem services (Milcu et al. 2013; Pohjanmies et al. 2017; Jaligot 2019). Services offered by trees can be understood by examining the emotional, sociocultural and symbolic meanings of trees (Satz et al. 2013). We consider that trees have significant importance to human living not only materially, but also emotionally. Understanding better what those intangible services are, and how they are formed, is essential for creating and sustaining physically and emotionally healthy, happy and meaningful environments. Trees have a great role in our societies, which this study aims to make visible.

### The material value of trees

Trees offer considerable economic benefits, as well as materials and food security (Pohjanmies et al. 2017; Przewoźna et al. 2022). Additionally, trees provide numerous ecosystem services in both urban and rural environments, including the regulation of air quality, noise, heat, pollution, and flooding, as well as the provision of materials and habitats that support biodiversity (Przewoźna et al. 2022). People perceive trees not only as resources to be exploited but also as entities with aesthetic (Dwyer et al. 1992; Tyrväinen et al. 2003) and recreational value (Hartig et al. 2003; Simkin 2020; Brito et al. 2022).

Urban trees influence the economy not only through their timber value but also by enhancing real estate values. Numerous studies have demonstrated that trees located on the properties, along the streets and in nearby parks and greenways increase the appeal of housing and, indirectly, the willingness to pay for it, reflecting the perceived value of the benefits that individuals expect to gain from the presence of trees (Orland et al. 1992; Jones et al. 2013; Giergiczny and Kronenberg 2014).

Trees, as integral components of urban green spaces have been shown to reduce stress, elicit positive emotions, and enhance concentration (Ulrich et al. 1991; Hartig et al. 2003; Simkin et al. 2020). The health benefits of visiting green areas are well-documented; moreover, research indicates that seeing trees from windows can provide similar health benefits on, for example, hospital patients (Ulrich 2002; Alerby and Engström 2021). Studies in environmental psychology has identified that the positive effects are particularly pronounced in favourite places in the nature (see, e.g., Ulrich et al. 1991; Korpela 2001). In addition to promoting self-regulation and cognitive restoration, these places are important for the building of attachment and identity (Korpela et al. 2001).

Benefits described here hold significant economic value, providing savings in communal costs, such as healthcare (Dwyer et al. 1992). While urban forest and tree management also have costs, estimating the overall monetary benefits of urban trees has proven challenging (Dwyer et al. 1992). Benefits include goods and services produced by urban trees, which are valuable to both people and urban ecosystems. While some benefits can be quantified in monetary terms, others are difficult to measure, or even perceive. These are referred to as immaterial, or intangible benefits.

### The immaterial value of trees

The intangible benefits of trees include social dimensions, such as creating desirable environments for quality everyday life and recreation (Dwyer et al. 1992) as well as fostering social connectivity and community stability (Wolf 2004). Socially, trees benefit people indirectly by increasing enjoyment in everyday life and enabling a stronger feeling of connection between people and their environment. In many human environments, trees are relatively stable and prominent elements often found in the immediate vicinity of homes, which makes them inviting objects to connect with and utilise for different purposes.

Trees can provide a variety of symbolic meanings (Altman 1994; Rival 1998), such as carrying universal mythologies and ontologies (Cusack 2011; Hall 2011; Harva and Anttonen 2019), national and local heritages (Haavio 1992; Jones and Cloke 2002), and identity building (Korpela 2001; Lummaa et al. 2023). They can also represent a variety of values, such as permanence and stability, trustworthiness, generosity, and fertility (Altman 1994).

The aesthetic value of trees increases enjoyment in everyday life and provides inspiration and a feeling of satisfaction. The sensory experience of natural beauty also encourages emotional and spiritual experiences (Rival 1998; Hall 2011). Trees are thought to offer space for spiritual experiences, as well as wisdom, assistance, and a safe space to reflect on emotions (Rival 1998; Abbot 2021).

Trees have the capability to create a feeling of connectivity and attachment to places, to nature, and to trees themselves (Jones and Cloke 2002). They have also been used in protests in political and environmental conflicts as a subject for protection (Davis and Jones 2014), a scapegoat, or an ally (Griffin 2008).

### Relations between humans and trees as other-than-human-persons

Many previous studies focus on a human-centred ecosystem approach, emphasizing the direct benefits of trees while objectifying them. This study challenges that view by focusing on interactions between humans and trees as entities with agency. We argue that benefits arise from variable and transient human-nature interactions that are both personal and sociocultural. To understand human dwelling as active engagements between the humans and the natural world, we focus on the manifold relationships between humans as persons and trees as other-than-human individuals.

The relationships between humans and nature have been explored in humanistic multispecies studies (Haraway 2008; Kirksey and Helmreich 2010). This framework emphasises recognizing “*other-than human-persons*”,—including animals, plants, microbes, and fungi—integral to the cultural world of humans, while also acknowledging humans as a part of the natural world. These entities are viewed as subjects with agency, intrinsic value and individuality. Alongside the “animal turn” (e.g. Haraway 2008) a branch of humanistic plant studies has emerged, encompassing various theoretical orientations (see e.g. Hall 2011; Gagliano et al. 2017; Woodward and Lemmer 2019; Nitzke and Braunbeck 2022; Slovic and Chou 2022). Common to these studies is a focus on textual materials (fiction, poetry, philosophy, etc.) (Hall 2011) or on ethnographic or historical analysis of specific groups' relationships with plants (see e.g. Lien and Pálsson 2021). However, research on human relationships with trees (or other plants) as other-than-human beings, has been limited, though it is increasing (see e.g. Tammi et al. 2020; Abbot 2021; Bergman and Östlund 2022; Barry et al. 2023). Studying trees as individual beings with sentience, consciousness, and capacity to communicate—and to be communicated with, reveals that they are not only objects for human action and reflection, but they posses their own interests and needs, which may not align with human desires. Thus, attention, recognition, gratitude, respect, and collaboration with the trees are essential elements of human-arboreal interactions (Abbot 2021) Tree relations are increasingly recodnized as cultural and philosophical issue in interdisciplinary environmental research, particularly within the environmental humanities. In our previous study (Vainio et al. 2024), we examined human-arboreal-relationships of biophilic interspecies friendship (Santas 2014), offering various possibilities for connection.

### Affordances in tree relationships

Following the *Biophilia hypothesis,* we understand that human beings are dependent on their natural environment in such a way that it creates a special bond, a love of life, and an affinity for other life forms. It also suggests that many human preferences are shaped by interactions with helpful features of the environment (Kellert and Wilson 1993). Hypothetically, those preferences are features that create an affinity towards trees. We understand that preferences are shaped by special features of trees (traits) and action possibilities that the traits create.

In this article, we focus on interaction in the relationship between humans and arboreal individuals and study affordances, the functional elements that trees offer. To do this, we implement *affordance theory* (Gibson 1966, 1979), which is central to many current theories of landscape preference (see e.g. Kaplan 1985; Summit and Sommer 1999). According to the original theory, affordances are considered as the possibilities, benefits, and harm that the environment provides for the perceiver. What trees have to offer, theoretically, shapes human attitudes towards trees. In the context *of biophilia hypothesis*, humans, like other animals, perceive the possibilities provided by their environment, especially features connected to their species-specific preferences and needs (Kellert and Wilson 1993). In addition to biological tendencies, humans also have preferences connected to their skills, lifestyles, personal history and cultural background. The core idea with affordances is that what is perceived depends on both the environment and the perceiver (Gibson 1979; Ingold 2011). Affordances can be material, such as a climbing possibility, wood, fruit, or a canopy providing shade, but they can be immaterial as well. Intangible, or immaterial, affordances can have sociocultural or psychological aspects, such as a place for recreation and inspiration, stress reduction, or a feeling of safety.

### Research hypothesis

Previously, we have found that human-arboreal relationships are related to the properties of humans and the preferred traits of trees (Vainio et al. 2024). In this study, our hypothesis is that traits formulate the means of interaction (affordances) between trees and humans. Our research is empirical, focusing on the actual relationships between people and trees, rather than solely cultural representations or meanings of tree relationships, as seen in critical plant studies or humanistic plant studies. In our study, we reached the microlevel, by asking the people who responded to our survey to think about their favourite tree-individual and to answer questions in the context of that tree. In this way, the study has sought to combine traits of ecology with a posthumanistically informed study of human relationships with nature. The study thus bridges the gap between humanistic and scientific approaches to understanding tree relationships as something that can be measured but also that requires cultural interpretation and understanding. To grasp all these aspects, including personal experiences and the effect of life history, we have chosen to focus on relationships and interaction between individual trees and individual humans. We conducted a survey on humans, trees, and their relationships in the Netherlands, which is one of the most densely populated European countries and where the availability of green areas and trees is limited.

The two primary questions quiding our research are (1) what kind of characteristics the relationships between people and trees are made up of and (2) how do these relationships manifest in practice? I addressed these overarching questions through a series of more detailed research questions: (i) What are the defining characteristics of human-arboreal relationships? Do the characteristics of humans and trees have a connection to the relationships that develop between them? (ii) What opportunities and benefits do trees offer to people, and what types of interaction does this create?

## Theoretical framework

### The original theory of affordances

American psychologist Gibson (1966, 1979) introduced the concept of affordance as an elemental part of his more general theory of visual perception, in which he examined animal-environment interactions. For Gibson, “the *affordances* of the environment are what it *offers* the animal, what it *provides* or *furnishes*, either for good or ill” (Gibson 1979, emphasis in original). Below, we use the word *provide* when referring to the affordances provided by favourite trees; alternatively, we use the word affordance directly. Consequently, affordances are understood to emerge from interaction with the environment (Gibson 1979), which is different from an understanding of the benefits as objective features of the environment (Chemero 2003).

The connection between sensory perception and material action is straightforward in the original theory: a perceiver does not need to initially think about the object and then consider possible actions (Caiani 2014; Jamone et al. 2018). In Gibson's original theory (1979), visual (sensory) messages from the material environment are directly interpreted as possibilities for material action, without more complicated mental processing (Caiani 2014). This approach is also how affordances are operationalised in many empirical studies. However, affordance theory allows for a wide range of interpretations—affordances, perception, and action can be understood in various ways (Kaplan 1985; Caiani 2014; Jamone et al. 2018). In empirical affordance studies related to the human-nature relationship and landscape preferences, visual messages from the environment often seem to directly afford many kinds of immaterial benefits, such as eliciting different emotions, and visual perception seems to be intertwined with cultural, social, and nonsensory personal processes (e.g., Kaplan 1985; Jokinen 2002; Ingold 2011; Rantala and Puhakka 2020).

Affordance theory has been applied in many different fields of research, such as psychology, anthropology, urban planning, design studies, landscape aesthetics, and technology development (e.g., Kaplan 1985; Gaver 1991; Summit and Sommer 1999; Ingold 2011; Nagy and Neff 2015; Jamone et al. 2018; Brito et al. 2022), where an analysis of an individual’s use or perception of different natural, constructed, or virtual spaces has been essential. For our study, affordance studies from the field of environmental (or ecological) psychology and landscape preference studies are central. These provide further evidence of the positive effects of trees (and other natural environments) on human well-being (see, e.g., Laaksoharju and Rappe 2017; Rantala and Puhakka 2020), but also point out that there are costs and harms involved (Dwyer et al. 1992). Previous studies indicate how relationships with arboreal individuals and trees, in general, are central to many aspects of human personal well-being and meaningful sociocultural life. This study will contribute by further developing a theoretical framework in which human-arboreal relationships can be studied.

### Our application of the affordance theory

**Fig. 1:**
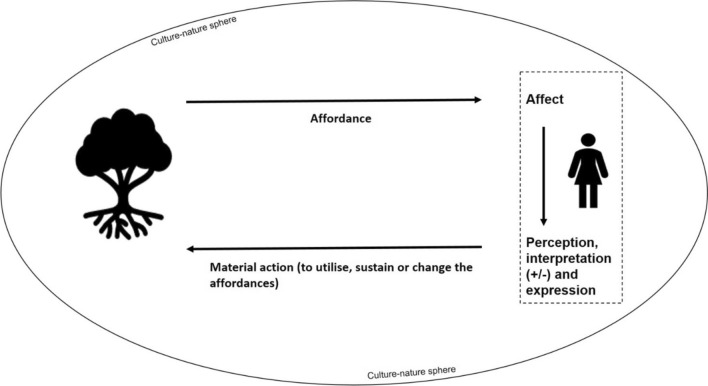
Theoretical framework for analysis

In this paper, we deviate from the original affordance theory by including immaterial benefits in the concept of affordance and by expanding from the original idea (Gibson 1966) of visual perception. In our theoretical framework, affordances are whatever benefits and possibilities favourite trees provide for human beings (Fig. 1).

In this study, we concentrate on the affordances as benefits provided by favourite trees perceived by humans. A human is affected by and perceives some of the affordances provided by the favourite tree. It depends on the individual whether an affordance is regarded as a benefit or a harm. Only after perception and interpretation can a human being express their experience of affordances. Perceptions and interpretations can also lead to material or immaterial activities that utilise, sustain, or change the affordance. The personal process is affected by nature and culture as an intertwined whole.

#### Emotional affordances

With our theoretical framework, we conceptualise how affordances are perceived and processed by a human being (Fig. 1). As affordance theory does not examine this process in detail, we add the concept of *affect* to our framework. There are several theories of affect, but we follow the original conceptualisation described in Spinoza’s Ethics (Spinoza 1677/2002, book 3). Spinoza (1677) defined affect as “*the capacity to affect and be affected*”. Affect precedes emotion, interpretation, and action. This locates affect in encounters in the world, rather than in the interiority of a psychological subject (Sedgwick et al. 1995), similarly to the concept of affordance, which also emphasises the priority of relationships in any meaning-making. Emotion is the interiorisation of affect towards psychological expression (Massumi 1995). Affect exists in a non-conscious 'zone of indistinction' or 'zone of indeterminacy' between thought and action (Massumi 2002). This also leaves space for unqualified and unrecognised affects (Massumi 1995).

Thus, humans observe all objects providing affordances, and simultaneously, these very same objects may also arouse affects that cannot be directly communicated or even conceptualised. The concept of affordance explains what the object is perceived to provide for the observer, while the concept of affect nominates a specific experiential or emotional state shift in the observer (Fig. 1). Understood as preceding what can be put into words, affect escapes any definition and cannot be defined in our analysis either. We include the concept in our framework, as it points to how our understanding of affordance is linked with emotions and is always limited by our capacity to express our experiences.

#### Immaterial activities

In our analysis, immaterial activities are also regarded as affordances, which differ from Gibson’s (1966, 1979) theory. We keep the definition of affordance as action possibilities but include immaterial activities such as thinking and feeling, thus incorporating the so-called *affective affordances* into our definition. Affective affordances have the capacity to activate, regulate, amplify, suppress, or otherwise alter our emotional states and experiences (Caravá and Scorolli 2020; Krueger and Colombetti 2018). In fact, our theoretical model assumes that there are no perceived affordances that could escape human values and emotions. This also implies that even strongly material affordances, such as the possibility of eating an apple, are associated with personal values and emotions, as well as the surrounding culture-nature sphere (Fig. 1). In addition to this kind of intertwining, affordances can also form sequences in which the perception and utilisation of one affordance can open new affordances (Gaver 1991). We also assume that some affordances elicit immaterial action, such as joy or sorrow, primarily or only, whereas some perceived affordances can be primarily material.

#### Culture-nature sphere

Finally, our theoretical framework proposes that the intertwined nature and culture around an individual strongly determine the kinds of affordances they have and how they are perceived and utilised (Fig. 1). In particular, the cultural and social dependency of affordance perception is often emphasised (e.g., Gaver 1991; Jokinen 2002; Rietveld and Kiverstein 2014; Rantala and Puhakka 2020) as social and physical environments set boundaries for behaviours, and frame beliefs and attitudes (Linder et al. 2022). Furthermore, much of contemporary scholarship on multispecies relationships has highlighted the entanglement and co-existence of humans and other species, their 'irreducible alterity and infinite connection' (Massumi 1995). Our culture-nature sphere suggests this entanglement and co-existence while retaining a certain conceptual difference between culture and nature, setting it apart from the more posthumanistic conceptualisations that seek to abolish the differentiation between culture and nature altogether (e.g., Haraway 2008).

*Affordance theory in this study* helped us consider how individual trees create specific possibilities for people. The concept of affordance is central to understanding the benefits or advantages that a particular tree offers to individual humans. While traits ecology focuses on the characteristics of individual organisms, such as trees, it does not explain the meanings or values of traits to people. Humanistic approaches, including plant studies and multispecies studies, delve into the meanings of plants for humans; however, such research has rarely examined the benefits of individual trees for individual humans. Therefore, the concept and theory of affordances were therefore needed to bridge the gap between the perceived characteristics (or traits) of trees and the experiences of benefits or values associated with these characteristics (or traits).

Affordance theory was implemented in our study to find the basis on which the interaction between humans and trees is built and how the interaction comes about. We were especially interested in discovering what the perceived benefits that trees offer to humans, which make them willing to form a continuous relationship with certain, specifically important tree individuals. To understand this, we collected different types of affordances: what the trees were providing, what humans were perceiving, and how they were utilising and sustaining them. In practice, affordance theory helped us to analyse the research material from the perspective of interaction and how material interaction was creating immaterial results, such as strengthening and expressing values, enabling emotions and connections, and creating memories.

## Material and methods

### Data

Our data (*n* = 158 responses) were collected using a survey (Online Supplementry Appendix A) distributed with a press release, performed with snowball sampling (Abbott and McKinney 2013), and commenced with our Dutch researchers. The snowball method allowed us to target people who have favourite trees, most of whom probably had some direct or indirect contact with Wageningen University & Research. Thus, our study is more a descriptive case study than a representative sample of the Dutch population. A non-response bias analysis was not carried out, as there were no data available to analyse how our sample group with favourite trees may differ from the wider population with favourite trees.

Survey questions were built in the Trees Near Us-project during data collection in Finland (Vainio et al. 2024). In the survey, we used a sensory approach (Rodaway 1994; Pink 2015) to gather information about the perceived tree traits. The goal was to conduct an interdisciplinary study combining approaches from trait-based ecology (Klausmeier et al. 2020) and multispecies studies (f ex. Kirksey and Helmreich 2010; van Dooren et al. 2016). We chose affordance theory for this separate study in the analysis phase to obtain additional results and a deeper understanding of the combined quantitative and qualitative survey results.

The questionnaire (Online Supplementry Appendix A) was divided into three sections that dealt with (i) the traits of the respondents (e.g., age, gender), (ii) the traits of their favourite trees (e.g., height, location, sensory features), and (iii) the relationships between the respondents and the tree (e.g., emotions, preferences, activities related to trees, length of the relationship, sustaining the relationship). Many of the questions described sensory features. Our hypothesis was that certain features of trees produced positive responses in people, i.e., provided them with certain affordances. To understand these affordances, we needed to know both about the features of trees and about how people understand and experience these features.

Despite this categorisation, many of the themes were covered in several sections. For example, the tree section contained questions related to the characteristics of favourite trees, and the human-arboreal relationship section included questions related to the tree characteristics that were of particular importance to the respondents. All sections contained closed- and open-ended questions. Closed-ended questions included multiple choice and single-choice questions on categorical and ordinal scales (Online Supplementry Appendix A).

The human-arboreal relationship section was emphasised in our analyses, as affordances emerge from interactions. Other questionnaire sections provided complementary information to determine whether types of people and trees were associated with particular types of human-arboreal relationships and the affordances therein. The survey questions related to the human-arboreal relationship mapped action possibilities, but also the benefits not specified as actions (Online Supplementry Appendix A).

### Analysis methods

First, the data from the closed-ended survey questions were arranged in binary form. The levels of the nominal- and ordinal-scale questions were treated as separate variables. Variables that occurred less than four times were excluded from the analyses. The three sections of the data—respondent traits, tree traits, and human-arboreal relationships—produced three binary data matrices. Respondent data included 80 variables, 89 tree data variables, and 61 human-arboreal relationship data variables (Online Supplementry Appendix B). The number and identity of the respondents (that is, the sample units) were the same (158) for the three data sets.

**Fig. 2:**
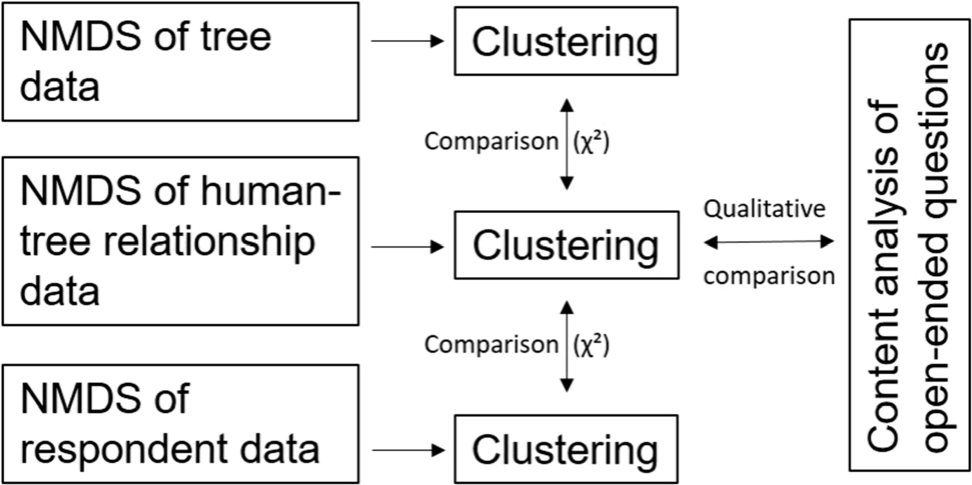
Analysis stages. Note. NMDS refers to multidimensional nonmetric scaling

Second, we used nonmetric multidimensional scaling (NMDS) to identify the main patterns of our multidimensional data (Fig. 2). In NMDS, the variables and the respondents are arranged in an n-dimensional ordination space based on their (dis)similarity (McCune and Grace 2002). In our case, the variables that were typically raised by the same respondents and the respondents who gave similar responses were placed near each other. The dimensions (axes) of the NMDS solution depict the main gradients in the data. The number of dimensions of NMDS was decided based on the stress value and the interpretability of the NMDS solution. The stress value is an indicator of how well the NMDS solution depicts the original data, with values < 0.25 deemed acceptable (McCune and Grace 2002). In the interpretation of these main gradients, we examine how variables and respondents were separated by the NMDS dimensions. We used the Bray–Curtis dissimilarity index as our distance measure. The analyses were carried out using the Vegan package (Oksanen et al. 2017) in R (R Core Team 2017).

A three-dimensional NMDS solution was selected for analyses of human-nature relationships and tree traits. The final stress value was 0.23 in both the human-arboreal relationship NMDS and the tree trait NMDS. Finding an acceptable NMDS solution was more difficult with the respondent trait data. We settled on a five-dimensional solution with a stress value of 0.16. In all three NMDS solutions, dimensions one and two depicted the interpretable main gradients. Thus, we concentrated on these two dimensions and the clusters created based on them. The exact positions of the variables and respondents of all included NMDS dimensions, as well as the results of k-medoids clustering, can be found in Online Supplementry Appendix B.

Third, the clustering tool of K-medoids in R (Reynolds et al. 1992; Schubert and Rousseeuw 2019) was applied to form clusters based on the NMDS dimensions for the three datasets (Fig. 2). The number of clusters and the number of NMDS dimensions used in clustering was decided iteratively, based on the interpretability of the clusters. We started with several (three or four) NMDS dimensions and four clusters. If the clusters were not easily interpreted, we then tested three clusters. If this did not help, we excluded an NMDS dimension and started again with four clusters. In all three cases, we ended up with a three-cluster solution based on the two first NMDS axes. These clusters were regarded the main types of respondents, trees, and human-arboreal relationships in our data. To obtain a more detailed picture of the content of the cluster, we investigated the occurrence of each variable within each of the three clusters of human-arboreal relationships (Online Supplementry Appendix B). Variable positions in the NMDS space were also re-examined in this investigation, which helped us to recognise the variables that were shared by two or more clusters. All clusters were assigned a name that summarised the cluster content beyond individual variables.

Fourth, the quantitative analysis continued with cross-tabulation of the human-arboreal relationship clusters with the respondent and tree clusters (Fig. 2). The difference between the observed and expected frequencies was tested with the *χ*^2^ test. To better locate significant differences—and because we were interested in positive correlations between clusters—we only tested whether cluster frequencies with > 30% positive deviation from the expected frequency within each cluster of human-arboreal relationships differed from the expected frequencies. This resulted in five *χ*^2^ tests. We classified p-values < 0.01 as statistically significant and values between 0.01 and 0.05 as nearly significant. The *χ*^2^ tests were conducted with Excel (Microsoft Corporation, 2013).

At the final stage, we examined open-ended survey questions through theory-driven thematic qualitative content analysis (Braun and Clarke 2006) (Fig. 2). We examined all open questions, but emphasised the question 'Why did you choose this tree?' (Online Supplementry Appendix B). The analysis was carried out by two researchers. In the first analysis stage, the researchers individually identified themes that depicted the content, without any predefined themes. After working through all open questions, the two researchers then compared their findings and identified the tree-related affordances perceived within the cluster. We established a difference between the core affordances that formed the core content of a relationship type and the secondary affordances that were present but were less central within a relationship type. The aim of this qualitative analysis was to reinforce, clarify, and, if needed, change the interpretations made in the previous quantitative analysis stages (Teddlie and Tashakkori 2009). Quotations were also collected to better represent human-arboreal relationships for the reader.

## Results

### Main variation and patterns in the data

#### Relationships between humans and trees

**Fig. 3:**
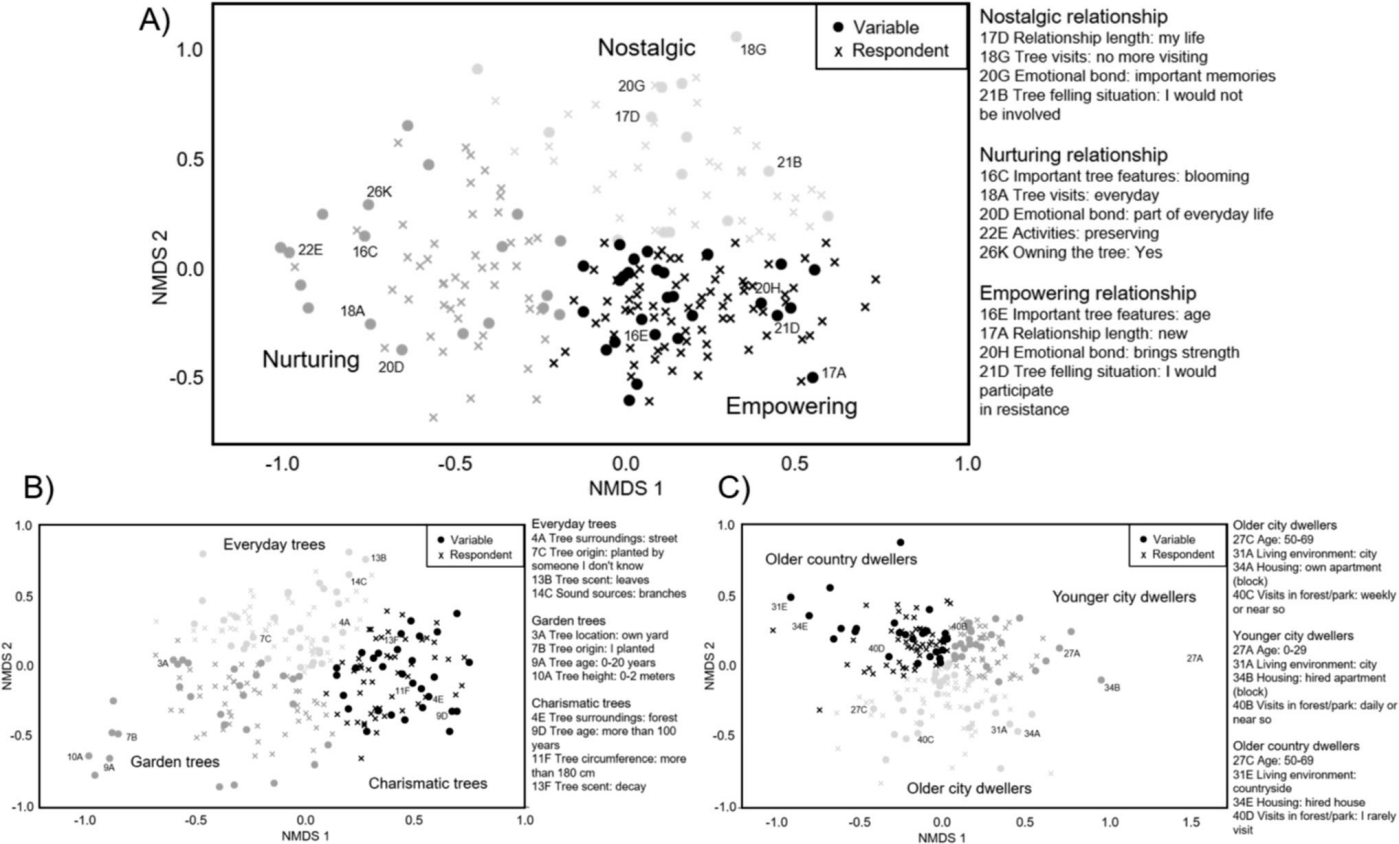
Results of nonmetric multidimensional scaling (NMDS) and k-medoids clustering. Note: Graphs present clusters (in black and grey, cluster names, and some example variables in two-dimensional NMDS space: **A** human-arboreal relationships, **B** tree traits, and **C** respondent traits

Three different clusters of human-arboreal relationship were identified in our analysis, each carrying specific types of perceived affordances that emerged from the human-arboreal interaction (Fig. 3), such as memories, companionship, and support. In the *nostalgic relationship* (*n* = 35), the favourite tree provided important memories, typically from the childhood of the respondent. In the *nurturing relationship* (*n* = 47), the favourite tree, usually located in the respondents’ own garden, provided companionship in everyday life, but also provided horticultural benefits, such as the provision of fruits or shade. Strength and support provided by the favourite tree was highlighted in the *empowering relationship* (*n* = 76). These three types of human-arboreal relationship are described in more detail in Sect. “The nature-culture sphere”. The types also reflected the main gradients of the NMDS solution: from nursing activities to nonmaterial connections along dimension one, and from present connection to the connection of memories along dimension two (Fig. 3).

#### Tree traits

From the tree trait data, we identified clusters of (i) everyday tree, (ii) garden tree, and (iii) charismatic tree (Fig. 3, Online Supplementry Appendix B). *The everyday trees* provided a visual, auditive, and even olfactory environment in the daily life of the respondent. Tree form, age, or size was not as important as the everyday presence. *Garden trees* were often planted by the respondent or someone close to them, so these favourite trees could also be small and young. The location was typically the respondent’s own yard or garden. As the name suggests, *charismatic trees* were usually old, large, and impressive, and the respondents highlighted the strength and support that these favourite trees conveyed to them.

Based on the cross-tabulation comparison between the clusters, *charismatic trees* were associated with the *empowering relationship* (*χ*^2^ (1) = 7.3, *p* = 0.007; Online Supplementry Appendix B) and *garden trees* with the *nurturing relationship* (*χ*^2^ (1) = 8.2, *p* = 0.004, Online Supplementry Appendix B). *The everyday trees* were most typical of the *nostalgic relationship*, although the result was not significant (*χ*^2^ (1) = 3.3, *p* = 0.071). Regarding tree taxa, oak and beech were typical *charismatic trees,* and birch was often reported as an *everyday tree* (Online Supplementry Appendix B), but otherwise it was difficult to perceive any strong associations between tree taxa and the three tree types. In total, 43 taxa were recorded in the survey (Table B6 in Online Supplementry Appendix B).

#### Human characteristics

Respondents were clustered as (i) *older city dwellers*, (ii) *younger city dwellers,* and (iii) *older country dwellers* (Fig. 3, Online Supplementry Appendix B). *Older city dwellers* were typically older than 30 years and there were an equal number of male and female respondents within this group. Many lived in their own apartment in a blockhouse and regarded themselves as urban people, and forests or parks were typically visited weekly. Many of the *younger city dwellers* were less than 30 years old, and there were more women than men in this group. The apartment house was also the most typical dwelling, but due to the younger age, apartment ownership was still rare. *Younger city dwellers* visited forests or parks almost every day and liked hiking and camping. The outbreak of the Covid-19 pandemic affected this group more than the others; during this time, forest and park visits increased or decreased. *Older country dwellers* were typically older than 30 years, and there were also more women than men in this group. They lived in a village or in the countryside and often had a row house apartment, owned or rented a house, or even a farm. *Older country dwellers* visited forests or parks less than the other groups, although forestry, gardening, and observing nature were typical for this group.

Based on the comparison of clusters, the *nostalgic relationship* with trees was typical for *younger city dwellers* (*χ*^2^ (1) = 4.3, *p* = 0.038; Online Supplementry Appendix B). *Older country dwellers* had a predominantly *nurturing relationship* with their favourite trees (*χ*^2^ (1) = 7.5, *p* = 0.006), while *older city dwellers* did not show a tendency to any specific human-arboreal relationship (see Table B5 in Online Supplementry Appendix B).

### Affordances in the human-arboreal relationships

#### Nostalgic relationship

In *nostalgic relationships*, the favourite tree played an important role in the childhood memories of the respondent (20G in Table B1 in Online Supplementry Appendix B; Table 1). During childhood, the tree and its surroundings had provided a good place to be alone, spend time, and relax, but was also an exciting and inspiring place to play, climb, and experiment with your own limits (id 33 in Table 1).

**Table 1:** Examples of a nostalgic human-arboreal relationship experience

	Nostalgic relationship
Id 33	I came across this tree because I went back in my memory and this tree was suddenly there again. It was the tree that I could climb very well, and it was very, very high. [–] I met the tree around 1960 when I was about 7 years old. You could climb in it and then jump from tree to tree. There were several trees in a row. It was kind of a Tarzan effect, jumping from one tree to another. [id33]
Id 91	This weeping birch is in the middle of my grandparents' garden on a lawn. It is in Germany, where I visit 1 to 2 times a year for a few days. It is my grandfather's pride that planted the tree. I used to play with it as a kid, but if you picked leaves, my grandfather would get very mad. Consequently, we did not do that, and all the grandchildren were careful with the tree. The tree is beautiful to look at, with moss on the branches, and is used as a parasol to sit under together. Now that my grandfather is gone, the tree reminds me of him. [–-] Now that my grandmother has been living in a care home for a few months, I don't know what will happen to the house and the tree. I would be sad if this tree was no longer there, but what do I do about it? I think any new resident can do what they want with their garden, but I hope they leave this beautiful tree and enjoy filtered light like my family used to. [id91]
id 158	I am still dreaming of it [id 158]

As a part of childhood, the favourite tree, or its memory, often provided a link to family history in a *nostalgic relationship.* Nostalgy could also persist in actions in adulthood provided that someone else would appreciate its advantages (id91 in Table 1).

In contrast to the other two types of human-arboreal relationship, no longer visiting the favourite tree was common in the *nostalgic relationship* (18G). In many cases, the tree was located at a considerable distance from the current living environment, although the favourite tree still lived on in the memories of the respondent. (id 158 in Table 1).

When asked if the favourite tree was threatened, many advocates of the *nostalgic relationship* reported that they would not do anything to save the tree or that they would understand that the tree must be felled (21A, 21B). This point of view, in addition to the low visiting activity of this group, was illustrative of how memories, specifically, were an important affordance. The association between younger age and *nostalgic relationship* (Table B5 in Online Supplementry Appendix B) raised a question regarding the change in the relationship. In our data, reporting an unchanged long relationship was particularly common for advocates of the *nostalgic relationship* (23K, 24C, 17D).

#### Nurturing relationship

In *nurturing relationships*, the favourite tree provided a range of possibilities for material action, especially connected with gardening or conservation. Raking (of leaves), control of pests and diseases, fertilisation and other soil improvement operations, physical support, careful monitoring, and crop harvest were all typical affordances in this type of relationship (22B–G, Table 1). Our data did not reveal to what extent these care-taking activities were perceived as benefits or harm. The tree was sometimes planted by the respondent, which was not typical of the other types of relationships (id 80 in Table 2).

**Table 2:** Examples of a nurturing human-arboreal relationship experience

	Nurturing relationship
Id 80	We live beside and under this tree and I am very happy that we came to live here 4.5 years ago. I did my best to develop an emotional connection with him (the tree), because initially when I came to live here 4.5 years ago, he was overwhelming. He stood in the gravel; we immediately improved the soil with minerals and oxygen by a very knowledgeable company. [– –] We took care of him so that we were able to extend his health and lifespan very much. [id 80]
Id 108	This tree is on our property, I see it every day, and when we are outside, I listen to the sound of the wind on its branches or leaves. I am always happy to see its buds swell in spring and smell that special poplar scent when the flowers fall off. It is the tallest and largest tree on our property; it is a striking tree. [id 108]
id 97	He stands in our backyard and provides fruit and shade, clean air, and is a nice hiding and breeding place for birds! Years ago, in spring, you would hear the magnificent buzz of hundreds of bees! That was great news. He has pears and beautiful blossoms. [id 97]

The nurtured tree was typically located in the garden of the respondent and was therefore an essential component of their daily life (18A, 20D, Table 1). In addition to caring activities, their nurtured tree provided the respondent with a place for relaxation and many delightful sensory experiences. Admiration of the beauty of the tree was often reported and when the tree was part of their daily lives, respondents noted how easy it was to follow its changing appearance over the seasons (16H, Table 1). For example, the moments of tree bloom and fruiting were especially important in the *nurturing relationship*, and animal life in the tree was often followed (16C, 16D, 16L).Interestingly, scientific curiosity was also connected with the *nurturing relationships* in our data. The favourite tree provided opportunities to observe and understand how nature works (id 97 in Table 1).

Under a hypothetical tree-felling threat, advocates of the *nurturing relationship* most often reported that they would simply prevent the felling (21F) or, if this was not sufficient, organise resistance to its felling (21E). In occasional cases—probably when the felling had an accepted reason, the respondents indicated a willingness to assist in the felling (21C). This point of view obviously reflected that tree owners have the potential to create a nurturing *relationship* (26K).

#### Empowering relationship

The strength and support provided by the favourite tree were emphasised in the *empowering relationship* (20H; Table 4). The tree was visited regularly, and this visit had restorative and empowering effects for the respondents in both happy and unhappy times of life (18B, 20E). Touching and other sensing of the tree were often emphasised in this type of relationship (16O, id 179 in Table 3).

**Table 3:** Examples of an empowering human-arboreal relationship experience

	Empowering relationships
Id 179	[– –] when I was sad, this tree comforted me and there I sat, I spoke to this tree because it had no judgment and there were no obstacles, I could be myself and felt that the branches took over my worries (comforting arms). [id179]
Id 182	The tree stands on the edge of a small moor that the tree overlooks. In the morning, when it is foggy, it looks very mystical. Because of this and because of the shape of the tree, I can imagine myself in another world. [–] It is a short asymmetric tree, not beautiful at first sight but strong, that has survived a moor fire. I can identify with the tree. Also, the form fascinates me, the squares of needled branches have something Japanese. The moor field is also the cemetery for pets (I live in a flat), so the tree also guards our animals. [id182]
id 93	I always see her on my way to the park when I walk the dog. The neighbourhood in which she is located is uninspiring, and when you suddenly see the tree, her beauty overwhelms you. She is so old, so scarred, so beautiful. She feels 'wise'. [id93]
- id 178	- This tree has been threatened with felling and the procedure is ongoing. I feel strongly in my own body when I think about the felling of this tree. To my own surprise. But it is the reason to fight against the (unnecessary) felling. [id178]

Charismatic trees, old, large and impressive, that stood out from their already scenic or, alternatively, unpleasant surroundings, were characteristic of the *empowering relationship* (16A, 16E, 16I, 20F in Table B4). Native oaks and beeches were common tree taxa (6B, 6E). Compared to the other two types of relationship, the *empowering relationship* was often considerably new, that is, a long common history was not necessarily needed in this type of relationship (17A, 17B). The advocates of the *empowering relationship* tended to have many favourite trees (23U), which illustrated their general tendency to admire charismatic trees.

**Table 4:** Core and secondary affordances for Human-arboreal Relationships

	Nostalgic relationship	Nurturing relationship	Empowering relationship
Core affordances	Memorising, having a companion when growing up, feeling connection to history/time, feeling connection to childhood/family/ friends/parents/ grandparents, playing, climbing, having a safe place, calming down, utilising shade, spending time, sitting, celebrating with family/ friends	Gardening and tree-care activities, having a companion in everyday life, coexisting, having a tree friend, enjoying (in general), enjoying beauty, utilising shade, relaxing, experiencing seasons, observing/recognising biodiversity, understanding nature, nurturing the tree, monitoring,, feeling happiness, material connection, sensory experiences	Empowerment, Feeling admiration, enjoying beauty, feeling connection to nature/universe/time/history/tree/ (person/family)/spiritual/mystical, sensing the tree, touching, hugging/leaning, sharing worries/feelings, self-reflecting, meditating, having a tree friend (the one that is greeted when met), recognising the intrinsic value of nature/ power of nature
Secondary affordances	Feeling admiration, enjoying beauty, sensing the tree	Feeling connection to family/ childhood/history, memories, worrying over a threatened tree, feeling sorrow because of a lost tree, feeling of permanence, feeling admiration,	Worrying over a threatened tree, feeling sorrow because of a lost tree, facing conflicts, participating in activism, seeing nature in the city, having a special place, forming a landmark/sight/local history/identity of place

Affordances within each of the three types of human relationships identified from open survey questions, specifically in answer to the question 'Why did you choose this tree?' (Online Supplementry Appendix B). The core affordances formed the main content of the relationship type. Secondary affordances were present but were still marginal in the type of relationship. Note that the same affordances could occur within different types of relationship

In the *empowering relationship* the favourite tree provided connections to something 'bigger' (20K). This could mean various things: we recorded connections to the tree itself, history, time, nature, departed people, and religious and mystical themes (id 182 in Table 3). The favourite tree was also commonly seen as a manifestation of the power of nature or its intrinsic value.

If the favourite tree were threatened by felling, advocates of the *empowering relationship* would be the readiest to defend the tree. They indicated a willingness to participate in organised resistance and to take the initiative themselves (21D, 21E). Otherwise, and like the *nostalgic relationship, the empowerment relationship* was not characterised by other care-taking activities (22A).

## Discussion

Through mixed methods analysis, we (1) identified three types of human-arboreal relationships, (2) explored the affordances that trees can provide, (3) examined how these affordances are perceived, and (4) determined the kinds of activities needed to sustain and utilise these affordances. Our findings could be useful in land use planning in urban contexts both in the Netherlands and in other parts of the world where trees are present in human surroundings.

### Affordances in human-arboreal relationships

Our results illustrate the diverse forms that human-arboreal relationships can take. *Nostalgic*, *nurturing,* and *empowering* relationships provide different material and immaterial benefits and opportunities (affordances) to humans. The types of human-arboreal relationships are reminiscent of the biophilic friendship categorization (Santas 2014), based on Aristotelian friendship categories. These friendships offer different types of connections—material, sensory, transcendent, and symbolic—depending on the action potential afforded by the specific trees involved (Vainio et al. 2024).

Affordances are based on the traits of the tree types that provide them (*everyday trees, garden trees, charismatic trees*) and the different needs, values, and skills of the human groups that perceive them (*older city dwellers, younger city dwellers, older country dwellers*). For example, if we look at the perceived possibilities for material action, there is a clear gradient from multiple possibilities in the *nurturing relationship* to no present-day possibilities in the *nostalgic relationship*. However, the latter is dependent on the past, when earlier, intensive material activity, often in childhood, was re-experienced through immaterial memorisation. It is well researched how children are creative in utilising the material action possibilities provided by natural environments and trees (Laaksoharju and Rappe 2017; Rantala and Puhakka 2020), and these experiences seem to have positive effects on having a rich human-arboreal relationship also at a later age. That finding is supported by research about nature connections, where previous activity has been the main factor for positive environmental sensing, valuation, and empathy (e.g. Sommer 1997; Linder et al. 2022; Barry et al. 2023).

Our results also illustrate how climbing and playing activities change to caring and touching as people get older. Simultaneously, people may exchange their favourite tree of childhood memories for a new favourite tree that reminds them of the previous one. The replacement of material interaction with intangible memorisation in the *nostalgic relationship* depicts the complex relationships that exist between affordances. For example, utilising one affordance could lead to the perception of another—the affordances become nested like a Russian doll (Gaver 1991). We see this in the *nostalgic relationship*, where the affordances utilised in childhood lead to memorisation in later age. Apparently, affordances can also be perceived and used in bundles; for example, obtaining a fruit harvest in a *nurturing relationship*. We researchers could treat the material harvest and the feelings of joy and pride about this harvest as separate affordances, but all come into being simultaneously, thereby making separation questionable.

People with a *nurturing relationship* lived their daily life with a favourite tree that provided affordances, such as the possibility to enjoy beauty and experience the changing of the seasons. There was no activity needed for utilisation of affordances. Thus, activity could also be materially, but not qualitatively, the same without the favourite tree. Gibson's (1979) theory already suggests that there can be intentional forms of (material) action other than the performative actions directly dedicated towards the use of affordances (Jokinen 2002).

The *nurturing relationship* effectively illustrates the various ways in which we can perceive the affordances, both actively and passively. For example, Finnish forest owners instinctively recognized certain affordances as part of their routines while also discovering new affordances during their walks in their forest (Jokinen 2002). Similarly, individuals with a *nurturing relationship* perceived the affordances that were consistently offered by their favourite tree, such as framing everyday scenery, but were also open to transient new affordances, such as the presence of birds or the vibrant autumn colours of the tree. The endless variability in nature continually presents opportunities for new affordances (Jokinen 2002). This type of exploratory activity is no longer possible within a *nostalgic relationship*.

Despite the apparent differences between these human-arboreal relationships, all were characterised by the positive effects that favourite trees had on human well-being. Individual trees seem to have the potential for similar positive effects on emotion regulation and cognitive restoration as natural environments in general (e.g., Ulrich et al. 1991; Hartig et al. 2003; Simkin et al. 2020). In addition to these benefits, we can expect favourite trees to have similar effects on place attachment and identity building as those exhibited by natural favourite places (Korpela et al. 2001; Bergstén 2018). Also, spiritual affordances in special trees can have a high potential for strengthening a person’s connection to their Indigenous heritage, such as the Sámi shaman Inga Enarsdotter (and potentially her successors) had in making offerings to her sacred tree (Bergman and Östlund 2022).

In the study of school gardens, where children cared for various plants and animals (Tammi et al. 2020), their interactions fostered what we describe as “nurturing” relationships. Here, children understood their wards from the perspective of friendships and relatedness, rather than perceiving benefits (Tammi et al. 2020). Their interaction interactions manifested in three distinct aspects: material and continuous efforts, emotional connections, and ethical and political considerations (Tammi et al. 2020). Similar patterns can be observed in human-arboreal relationships, where both material and immaterial affordances are utilized. Nurturing trees represents a material effort, while emotional connections contribute to relationship formation. Ethical considerations involve expressing values or engaging in political actions, such as protection or resistance against felling the trees.

Previous studies (Summit and Sommer 1999) have found that affordances, such as climbability, are associated with tree preferences and contribute to the attractiveness of certain trees. Research on attitudes toward street trees worldwide has (Sommer et al. 1997) revealed that acacia-like tree shapes with broad canopies, canopies—offering shade, short trunks, and relatively large sizes, are highly preferred for Studies about tree shape preferences suggest that the appeal of trees is not solely based on  geometry but rather on their natural forms (Sommer 1997). In this context, our results indicate affection is not merely about the tree traits but rather about the action possibilities that they provide. Consequently, the utilisation of material affordances yields additional immaterial benefits, such as a sense of place connectedness (Laaksoharju and Rappe 2017), avenues for spirituality (Bergman and Östlund 2022), feelings of friendship and connectivity (Tammi et al. 2020), and empathy for plants (Barry et al. 2023), all of which may contribute in pro-environmental behaviour (Linder et al. 2022).

### The nature-culture sphere

Our theoretical model suggests that the intertwined nature and culture—the culture-nature sphere—can influence affordances. In this context, we found that urban and rural environments provided different advantages and the potential to sustain and use them. For instance, *nurturing relationships* were more common in rural settings. The environmental context (wild, countryside, suburban, urban) has been shown to impact preferences for trees (Summit and Sommer 1999). Additionally, individuals' life stages and their socio-economic status affect their willingness to support trees: we observed that older and wealthier individuals  exhibited significantly different potential (compared to the other two groups) to gain, sustain, and change their preferred tree affordances due to their wealth and tree ownership. This phenomenon has also been evident in multiple choice experiments assessing the valuation of street trees (e.g. Orland et al. 1992; Zhang et al. 2007; Jones et al. 2013; Giergiczny and Kronenberg 2014).

We were curious to find out how the biophysical environment, particularly tree density, influences relationships with trees.  Previously, we conducted a similar survey in Finland, one of the most forest rich countries (Vainio et al. 2024), and we now aimed to compare the results with those from the Netherlands. Surprisingly, we did not observe significant differences in the human-arboreal-relationships. However, some variations were noted among different  tree and human groups. The scale of affordances was comparable, but the sample collected in the Netherlands placed greater emphasis  on environmental values. This may be attributed to the snowball sampling method used.  However, previous studies have provided evidence that a high tree density significantly enhances satisfaction with landscapes featuring urban trees and increases the willingness to protect these trees. Individuals living in proximity to trees place greater importance on various attributes of trees. Therefore, valuation of urban forests is not only connected with attitudes, knowledge and experience, but is also influenced by the current condition of the landscape itself (Davis and Jones 2014). This finding emphasizes the importance of considering the environment within the culture-nature-sphere in our theoretical model. Therefore, affordances are social, flexible, and context-dependent (Borghi 2021).

### The agency of trees, agency of humans in relationships

We propose that when humans form relationships with trees, it is not tree traits that are significant, but rather the affordances they provide. Affordances refer to the  action possibilities, and it is through action that the potential for a relationship is established. Trees are both acted upon and act upon their environment. Trees have different ways of utilising their agency, with their natural processes demonstrating both predictability and their surprising creativity. Additionally, trees possess agency through non-reflexive actions; they engage in the socio-ecological world as entities that are both being and becoming alongside humans. They have the ability to offer creative potential to humans and generate human emotions and emotional reactions (Jones and Cloke 2008). When humans consistently utilize the affordances from the same tree over time, the relationship strengthens, and ultimately a tree can become the Tree, a significant entity, referred to as arboreal person of importance. Interaction stemming from a sense of ”we-ness” (Franz and Mayer 2014), in which individuals perceive themselves as a part of nature (Schultz 2002), allows the Tree become a part of the cultural sphere. It is the relationships among various species that ultimately shape our multispecies reality.

Friendship and companionship are affordances that trees can offer. They fulfill the desire for togetherness and decrease the phenomenon of loneliness. The possibility of social interaction and multispecies care can also be seen as an important affordance. (O’Brien et al. 2014; Tammi et al. 2020). The meaningful relationships do not exclusively need to be with other humans; they can be between humans and other-than-humans, such as a tree (Alerby and Engström 2021; Vainio et al. 2024), dog (Haraway 2008), or a bird (Kavesh 2021). Affective and embodied encounters create a sense of belonging to the world. Connectedness with an other-than-human can enable the possibility to explore inner emotions, shape social choices, and lead to the discovery and reflection of the inner self (Kavesh 2021). Connection to an animal or a tree, enables people to temporarily forget their frustrations and create self-awareness. In cultural plant studies the empowering effects of trees on people, the role of tree care in cultures and the meanings of memories associated with trees are well known (Ryan 2017; Moe 2022; Nitzke and Braunbeck 2022).

In the survey data collected in the Netherlands, some people described their favourite trees as a unique beings with their own character. In the survey, they could use personal pronouns”ze = she” and”hij = he” (Dutch/English) when referring to “their tree” (Tables 2 and 3). In our other study of tree narratives in Finland (Lummaa et al. 2023), we found plenty of examples about experiences of trees as “friends”, “companions” and “fellow sufferers”. However, in the Finnish data gendering was not evident due to the language difference, since there are no separate pronouns for masculine and feminine individuals. Narratives help people structure and process their feelings and experiences about trees and their relationship with a particular tree. Narrative structuring helps to strengthen the connection with the tree, but also to distinguish the special qualities of the tree (Ryan 2017; Moe 2022; Nitzke and Braunbeck 2022).

Narratives also have a social dimension: they allow people to share their arboreal relationships with other people (Ryan 2017; Szczygielska and Cielemęcka 2019). In this way, the companionship and empathy felt towards trees can be strengthened into a communal force (Weber 2021). Acknowledging tree personhood can also be a choice representing environmental values or certain views of the world. The concept of plant personhood is also known in Indigenous discourses, where other-than-humans have an important role in kin relationships, where humans, animals, and plants (as well as spirits, etc.) are equal beings in the world (Barry et al. 2023). Indigenous cultures are not the only ones to see plants as having agency, as the idea is also rooted in European pre-Christian and traditional belief systems (Hall 2011).

Sense of oneness with the natural world is needed not only for human well-being, but also to create environmentally protective behaviour (Whitburn et al. 2019). Having a relationship with a tree is related to nature-positive values and involves actions to nurture the tree and nature altogether in general. According to previous studies (e.g., Brenguer 2007), a personal connection with other-than-human nature is the basis for feelings of empathy towards nature. Empathy therefore, is the key to provoking pro-environmental attitudes and behaviours towards other-than-human life.

### Implications for urban planning

Tragically, the emotional importance of a favourite tree can sometimes be noticed only after the tree has been threatened or felled, which can lead to conflict. The importance of different trees for local communities or individuals is difficult to interpret without inclusive planning, and in this decision-making, both tangible and intangible benefits must be considered (Jones and Cloke 2002; Przewoźna et al. 2022; Vainio et al. 2024). Some trees with local, natural, sacred, or historical value have been protected by law. Protection is a sign of value, which is both undeniable and visible—it allows many to understand and appreciate the affordances provided by a certain tree. Affordance studies typically advise land use planners and policymakers to provide people with diverse natural environments and natural elements (O'Brien et al. 2014; Laaksoharju and Rappe 2017; Rantala and Puhakka 2020; Brito et al. 2022). This diversity ensures a rich array of affordances for all, as individuals exhibit different tendencies in the perception of affordances and the capacity to use them. Based on our results, it is easy to agree with this policy advice. Furthermore, urban and rural settings differ both the functionality of trees, and the decision-making processes (Przewoźna et al. 2022). We found that rural landowners possess the most influence in decisions regarding the future of these trees.

In urban areas, decision-making is more complex since it is difficult to estimate, without inclusive planning processes, which trees are somebody’s favourites. However, humans seem to have concurring preferences for the aesthetic, species, shape, and size of trees (Summit and Sommer 1999; Przewoźna et al. 2022), which suggests a tendency to perceive useful affordances for human survival (such as shade, shelter, climbing possibilities, and fruits). The benefits associated with trees become increasingly valued as trees are more and more confined to green spaces in an overall urbanised landscape (Giergiczny and Kronenberg 2014). Urban forest resources have been understood to have a broad contribution to the economic and emotional vitality of the city and neighbourhood (Dwyer et al. 1992). Ecosystem services (cooling temperature, influencing wind, humidity, rainfall, air quality, etc.) that trees offer also have an impact on human health, happiness, and well-being of residents, which is difficult to reflect in monetary terms. There are material and immaterial benefits, but there are also both material and immaterial harms (such as costs, maintenance, feelings of danger, accidents, and messiness) (Dwyer et al. 1992). The main challenge in urban tree care is balancing benefits and harms, and there are also contrasting needs and preferences for urban trees. In this process, not only societal affordances but also  personal affordances such as interspecies relationships, should be considered. It is noteworthy to address that the benefits attributed to individuals can also have larger impacts on society: feeling connected with the nature around a person creates a stronger sense of community, a relatedness to local places, a decrease in crime and violence, and an empowerment to take environmental responsibility (Dwyer et al. 1992).

Some cities have identified themselves as “tree cities” (such as Joensuu in Finland, the city where this study originates), specifically focusing on the vitality, image and attractiveness which tree-rich city landscapes can offer. Trees are maintained professionally, but in collaborative cooperation with residents, for example running public surveys before tree maintenance in neighbourhoods. This can also be seen as a cost-saving measure as residents are actively perceiving the trees around them and participating in planning by requesting tree removal, saving or maintenance based on their preferences on sustaining the benefits that the trees offer them. However, since people value nature in diverse and often conflicting ways, promoting pro-environmental behaviors and simple solutions to environmental conflicts can be challenging (Raatikainen et al. 2023).

### Methodological considerations incorporating a more-than-human perspective

In this study, we primarily focused on the perceived affordances presented to respondents in the questionnaire. Although we included an open-ended question about their favourite tree, it was not the first question posed. Consequently, we cannot determine the extent to which we influenced respondents' perceptions of possible affordances.

The survey method employed did not allow us to capture the immediate moment of sensory perception; thus respondents had to rely on their memory. Additionally, we did not inquire about the social dimensions of human-tree interactions; however these aspects were spontaneously mentioned bye respondents in their open-ended answers, specifically regarding *nostalgic relationships*. Future studies would benefit from evaluating how the inclusion of an open question or interview format—allowing respondents to freely describe their relationship to their favourite tree rather than selecting from a predetermined list of potential affordances. We also acknowledge that the sample collected in the Netherlands is biased, as it predominantly includes individuals with strong environmental values.

We suggest that Gibson’s (1979) affordance theory can provide valuable insights into cultural ecosystem service if it expands, beyond material benefits to include immaterial action possibilities. This theory could clarify how cultural ecosystem services emerge from human-nature interactions. The nostalgic, nurturing, and empowering relationships identified in this study enhance existing research on human-arboreal relationships in ecosystem services research and humanistic plant studies.

Our goal was to challenge the human-centered and benefit-oriented ecosystem approach by focusing on interactions within relationships rather than connections between humans and trees. While complete avoidance of human-centrism is impossible, we propose our model of affective affordances as a framework for studying interactions between humans and more-than-humans, allowing for the integration of more-than-human perspectives.

Incorporating a more-than-human perspective into theoretical frameworks highlights the interdependence between humans and non-human entities. Research methodologies must consider the needs and impacts on non-humans as well. 

This might include examining how human care and protection enhance the health and growth of trees, thereby supporting biodiversity and ecological networks.

Data collection poses challenges, as we cannot directly obtain responses from non-human entities. Instead, we gather human experiences and ecological assessments to understand reciprocal interactions. 

Analysing the results from a multispecies perspective reveals how emotional bonds between humans and trees lead to beneficial actions, emphasising the importance of fostering connections that support both human well-being and ecological health in urban planning.

### Recommendations

Lastly, we advocate for a paradigm shift that emphasizes dialogic interaction over the mere benefits provided by trees. It is essential to shift focus from benefits offered by trees towards dialogic interaction. What are their affordances for other species? What are their needs? How can we humans offer some benefits for them in our interaction? Dialogic interaction creates respect towards other-than-human persons, and more commonly, towards nature (Hall 2011). Interaction is affordance which is proved to enhance environmentally responsible values and behaviours (Berenguer 2007). This is something which is needed in contemporary Western societies. Consequently, urban planning can significantly influence outcomes beyond the mere creation of aesthetically pleasing environments; it can facilitate biospheric restoration and promote respectful engagement with nature. Replacing instrumental relationships with social relationships characterized by care and solidarity is an ongoing endeavor aimed at dismantling the human-nature dichotomy (Hall 2011). Working closely with individual plant entities, we have the potential to transform our perceptions of nature.

In order to effectively plan greenspaces that encompass a diverse array of affordances, it is imperative to challenge the established Western perspectives regarding trees, plants, and nature. The prevailing Western attitude towards flora has often been characterized by a hierarchical view, wherein trees and plants are primarily regarded as resources for human exploitation and well-being (Hall 2011). We advocate for a paradigm shift that recognizes trees as entities with agency, akin to other-than-human persons, capable of interacting with humans and other animals. This recognition fosters connectivity and interaction between humans and trees, thereby facilitating dialogical engagement that cultivates social relationships essential for moral consideration of plants. The objective extends beyond merely creating more accessible green spaces; it aims to enhance interactions between humans and nature, thereby reinforcing a sense of connectedness with the natural world and promoting environmentally responsible values and behaviors.

## Conclusions

We discovered the connections between perceived tree traits and human characteristics that facilitate the emergence of human-arboreal-relationships. We identified several affordances that created connections between favourite trees and human individuals, allowing us to understand how these connections manifest as both material and immaterial actions to utilise and sustain those affordances.

The affordance framework, as applied, offers a plausible explanation for the emergence of human-arboreal relationships and can be utilised to examine the diverse range of qualitatively different benefits and interactions that these relationships encompass. However, the common application of this theory focuses on the affordances that nature provides to humans, maintaining a human-centered perspective. This approach also applies when we analyse the affordances that favorite trees offer to people. In an affordance-inspired analysis of the sustainable use of nature, we also encourage for the visualisation of the potential affordances that humans provide to trees. Humans and trees coexist and their lives are intertwined, and it is essential to recognize that humans are not, and should not be, the only species that derive benefits from these relationships.

Our findings suggest that the perceived quality of urban trees improves when opportunities exist to form different kinds of human-arboreal relationships and foster a connection with nature. Daily sensory enjoyment near one’s home, empowerment of multi-species companionships, and the potential for symbolic attachment through memories, spiritual experiences, and connections to specific places are essential components for human emotional well-being. These experiences should be accessible to everyone through the  inclusive planning of urban green spaces. Furthermore, the emotional significance of both urban and rural trees should overlooked; rather, it should be valued and preserved.

## Supplementary Information

Below is the link to the electronic supplementary material.

Supplementary file1 (PDF 1059 KB)
