# Supplementary material for: Green companions: Affordances of human–tree relationships
Source: Ambio. 2025 Jan 27;54(5):850–68. doi: 10.1007/s13280-024-02098-1 (PMC11965079; doi:10.1007/s13280-024-02098-1)
Supplement: Supplementary file 1 — Supplementary file1 (PDF 1059 KB) [file 13280_2024_2098_MOESM1_ESM.pdf]

Ambio

Supplementary Information

This supplementary information has not been peer reviewed.

Green companions: affordances of human-tree relationships

Kaisa K. Vainio [a, b], Tuomo Takala [a], Juul Limpens [c], Karoliina Lummaa [d], Aino Korrensalo [e], Aleksi Räsänen [f], Eeva-Stiina Tuittila [a]

[a] School of Forest Sciences, University of Eastern Finland, Joensuu, Finland

[b] Department of Geography and Geology, University of Turku, Turku, Finland

[c] Department of Environmental Sciences, Wageningen University, Wageningen, The Netherlands

[d] Department of History Culture and Art Studies, University of Turku, Turku, Finland and BIOS Research Unit, Helsinki, Finland

[e] Department of Environmental and Biological Sciences, University of Eastern Finland, Joensuu, Finland and Natural Resources Institute Finland, Joensuu, Finland

[f] Natural Resources Institute Finland, Oulu, Finland and Geography Research Unit, University of Oulu, Oulu, Finland

Appendix A. Survey questionnaire

Do you have a tree – friend?

What kind of relationships do people experience with trees? Which tree attracts you and why? Can a tree have an emotional value? How do we take care of trees important to us?

The research project 'Trees Near Us' explores what makes one tree more important than others. We would like to find out what people feel for trees, how these connections form and how they are expressed in everyday life.

The research takes place in both Finland and the Netherlands. Finland and Netherlands represent contrasts in forest cover while still sharing a lot of similar cultural values. This combination makes them an interesting pair to compare how people feel about trees in their environment.

'Trees Near Us' investigates the relationship between people and trees through science and art and is being led by the University of Eastern Finland in partnership with Wageningen University (the Netherlands). The team encompasses researchers from forest ecology, literature, and cultural anthropology, as well as professionals in the sound and photographic arts.

We use an online questionnaire to select representative cases of tree-human relationships. These cases are complemented with stories about trees from interviews. We approach the subject through contrasts. We look at trees in old and young urban culture and the differences in the relationship between the metropolitan area, small towns, and the countryside, in both the Netherlands and Finland.

With this research we increase the understanding of the cultural value of trees. This knowledge can be applied in urban and rural management of green areas as well in the forest planning,

The research is funded by the Finnish Kone Foundation and takes place between 2019-2023.

Tell us your story!

We would like to hear about your relationship with trees, also if you have no special feeling for trees at all. We are looking for respondents from people of all ages and backgrounds across Netherlands and Finland.

Visualize a tree that is important to you. If you have several important trees in your life, choose one. The tree can live, be dead, or no longer exist. You can fill in the form multiple times to tell us about all your favourite trees. It takes about 20 minutes of your time per tree.

Your information remains confidential. Read more about how we protect your privacy in the Research Bulletin.

Consent to participate in the study.

Participation in this study is voluntary. Replies are processed anonymously. The personal data or contact details of the participant will not be disclosed to third parties. Children under the age of 16 need permission from their parents or guardian to participate.

You may withdraw your consent to participate in the study at any time. However, the information provided until that moment can still be used as part of the study data. The information provided will be treated confidentially. The survey material will be archived in the Finnish Forest Museum Lusto archive for further research. Read more about the processing of personal data in the Research Bulletin.

1. **Consent**

I agree to participate in the research and give my consent to use the information I provide as described in the Research Bulletin. If I'm younger than 16 years, I have my guardian's permission to give my contact information.

- Yes. I agree to save my information.

Important tree

Think of an individual tree which has special significance to you and answer the questions. If you have several important trees, choose one of your favourites. The tree can be alive, dead, or you can choose a tree that no longer exists. If you wish, you can fill in the form several times and tell us about the different trees which have specific importance for you.

2. Is there any tree-individual important to you?

- Yes, some trees are much more important to me than others.
- No, all there are no trees that are much more important to me than others.

[If answered “no”.]

3. You do not have a favourite tree. Could you instead please describe your relationship to trees in general? (Open field)

It seems that you do not have a favourite tree in your life currently. In this case we are still interested in your general relationship with nature. You are directed to last part of the survey.

Characteristics of the tree

Think of an individual tree which has special significance to you and answer the questions. The questions below explore in which way your tree distinguishes itself from others.

4. Does your special tree still exist? (Please select one option that best describes the situation)

- The tree exists and is alive.
- The tree is dead, but its trunk has remained.
- The tree is gone.
- The tree has been felled or has fallen, but the wood material is in use by me or someone close to me.
- The tree has been felled or has fallen, and the wood has been taken somewhere for use.
- I do not know.
- Other, what?

If the tree you mentioned no longer exists, please answer the following questions by thinking of the tree as you remember it.

5. Where is the tree located? (Select the option that best describes the situation)

- In my home yard
- Close to my current home
- In or near my former home yard
- In the yard of a friend / relative
- Near my summer place
- Nearby or along the way to my work / school
- Elsewhere in my hometown
- Abroad

- Elsewhere - Please describe where.

6. Is the tree located in your current hometown?

- Yes
- No

7. In what kind of surroundings is the tree located? (You may choose several options)

- Along the street
- City Park
- Backyard
- At the forest edge
- In the forest
- On a hill
- By the water
- On a peatland
- On an island
- In another place - Please describe were

8. What type of tree is it?

- Evergreen tree
- Deciduous, broadleaved tree

9. What kind of tree is it?

- A native tree
- An exotic tree
- A fruit tree
- A nut tree.

10. To which species does the tree belong?

- Black locust
- Apple tree
- Beech
- Birch (birch or red birch)
- Goat willow
- Pine
- Oak
- Alder
- Maple
- Ash
- Maidenhair tree
- Hazel
- Elm
- Juniper
- Cherry tree

- Knotted willow
- Larch
- Rowan
- Linden
- Horse chestnut
- Pear tree
- Plane
- Aspen
- Plum tree
- White Willow
- Fir tree
- Chestnut
- White weeping willow / Glaucous weeping willow
- Bird cherry
- Walnut
- Willow
- I do not know.
- Other – Please describe.

11. Has the tree been planted? (Select the most descriptive option)

- No, the tree has established itself spontaneously.
- Yes, I planted the tree myself.
- Yes, the tree has been planted by someone close to me.
- Yes, but I do not know by whom.
- I do not know whether the tree has been planted or has established itself naturally.

12. Is the tree standing alone or in a group?

- Standalone
- Group

13. What is the approximate age of the tree? (Or what age was it when felled)

- 0 - 20 years
- 20 - 50 years
- 50 - 100 years
- over 100 years
- I do not know.

14. How high do you estimate the tree to be? (Note that your answer may depend on your height or the height of the buildings near the tree. Select the most fitting description.)

- The tree is my height or shorter (0-200 cm)
- The tree is taller than me (150-300 cm)
- The tree is at the height of a house or slightly higher (3 m-9 m)
- The tree is at the height of a 2-5 floor building (9 m-20 m)
- The tree is higher than a 5-floor building (20-30 m)

- The tree is very tall (over 30 m)

15. How thick is the tree trunk? (Girth measurement of the tree from your chest height). (Select the most fitting description)

- Thickness equal to finger (3-10cm)
- Thickness equal or smaller to my wrist (10-20cm)
- Thickness equal to a forearm (20-40cm)
- Thickness equal to a thigh (40-80cm)
- Thickness equal to my body (80-150cm)
- My arms reach around the tree (150-180cm)
- The tree is thicker than my arms reach (180cm+)

16. What is the autumn colour of the tree's leaves? (Select the most descriptive option)

- Same colour all year round
- Pale colouring
- Bright yellow
- Red or orange
- Multi-coloured
- The leaves / needles are green when dropping.
- I don't know.

17. Does the tree have a specific scent? (You can choose several options)

- No scent
- The scent of bark
- The scent of leaves
- The scent of flowers
- The scent of twigs
- The scent of resin
- The scent of decay / autumn
- I don't know.
- Something else - Please describe. [Open field]

18. Does the tree make a sound? (You can choose several options)

- No special sound
- The creak of the trunk
- Rustle of the leaves
- Shivering branches
- Animal / insect sounds
- I don't know.
- Something else - Please describe.

19. What is the shape of the tree trunk? (You can choose several options)

- Straight trunk
- Branched trunk
- Knots on the trunk
- Trunk with regular shape
- Crooked trunk
- Thick trunk
- Thin trunk
- Lots of twigs on the trunk
- Few twigs on the trunk

The relationship between human being and the tree

Continue thinking of the same individual tree and answer the following questions. The questions explore preferences and relationship to specific tree.

20. What qualities do you particularly like about this tree? (You can choose several options)

- Circumference (size)
- Height
- Flowers
- Fruits / berries / pinecones
- The age of the tree
- Colour of leaves / needles
- Shape of leaves / needles
- Autumn colouring of leaves / needles
- Shape of the trunk
- Location or the shape of branches
- Bark or surface of the trunk
- Tree-dwelling animals / birds / insects
- Special location
- Scent
- Sounds of the tree
- No pleasant features
- Other quality – Please describe.

21. How long have you had a special relationship with this tree? (Select the most descriptive option)

- Quite recently
- Several years
- For several decades
- All my life
- Throughout the whole life of the tree

22. How often do you visit this tree? (Choose the most descriptive option. If the tree no longer exists, choose “I don’t go to the tree anymore)

- Almost every day
- Weekly
- Monthly
- Several times a year
- Once or a couple of times a year
- Less than once a year
- I don’t go to the tree anymore.
- I don’t know.

23. What do you usually do near this tree?

[Open field]

24. What kind of emotional bond do you have to this tree? (You can choose several options)

- I have no emotional bond to the tree.
- This tree came to my mind for some reason.
- I see the tree often and pay attention to it
- The tree is part of my everyday life.
- Seeing this tree brings me joy.
- I admire this tree.
- There are important memories associated with the tree.
- I turn to the tree to gain strength.
- This tree is like a friend to me.
- I share my joy and sorrow with the tree.
- The tree represents something bigger than me - Please explain what.
- Other - Please describe [Open field]

25. What would you be prepared to do if someone would propose this tree should be felled? (You can choose several options. If your tree no longer exists, choose what you did or would have done in this situation?)

- I do not know.
- I would do nothing; I would understand the situation.
- I would not like to be involved.
- I would help felling the tree or I would fell it myself
- I would attend a discussion, appeal, or a demonstration organized by somebody else.
- I would act against felling the tree myself by talking with the person(s) responsible, initiating a discussion, appeal, or demonstration.
- I would prevent the felling of the tree by any means.

26. Do, or did, you take care of the tree, or the nuisance caused by the tree? (You can choose several options)

- No care of the tree
- Leaf raking
- Cleaning of other debris caused by the tree.
- Harvesting of fruits or other crops

- Preserving the tree (support, tying, etc.)
- Protection of the tree from animals or insects
- I harvest the wood of the tree.
- I prevent the felling of the tree.
- Something else - Please explain [Open field]

27. Has your favourite tree changed during your lifetime?

- No, my favourite tree has always been the same.
- Yes, my favourite tree has changed.
- I have many favourite trees.

28. Was it difficult to select one tree for this survey?

- Yes, it was hard to pick one tree out of many.
- Yes, it was difficult to come up with a suitable tree.
- No, I have one clear favourite tree.
- No, this one easily came to my mind among several potential trees.

29. Why did you choose this tree? Does the tree have a story? Would you like to talk more about this tree and your relationship with it?

[Open field]

30. Do you own or does someone close to you own this tree?

- Yes
- No
- I do not know.

Background of the respondent

This section collects information about the survey respondents.

31. Age of the respondent

- 0 - 17
- 18 - 29
- 30 - 39
- 40 - 49
- 50 - 59
- 60 - 69
- 70 - 79
- 80 - 89
- 90+

32. Gender

- Female

- Male
- Non-binary

33. Current place of residence? (Province)

- Drenthe
- Flevoland
- Friesland
- Gelderland
- Groningen
- Limburg
- North Brabant (Noord-Brabant)
- North Holland (Noord-Holland)
- Overijssel
- Utrecht
- Zeeland
- South Holland (Zuid-Holland)

34. Current place of residence? (Please enter postal code)

35. In what kind of environment do you live?

- Big city
- Suburban area
- Small town
- Village
- Rural area

36. Place of birth (fill in the name of the municipality)

37. Do you live in the same municipality where you were of born?

- Yes
- No

38. Do you think of yourself more as a urban or a rural person?

- Urban person
- countryman / countrywoman
- Both
- Neither

39. What kind of place do you live in?

- Apartment building as an owner
- Apartment building as a tenant
- Row house as owner (row house or duplex)
- Row house as a tenant
- Detached house as an owner.
- Detached house as a tenant.

- In a dormitory / service unit / nursing home
- Other - Please describe.

40. How many times in your life have you moved to another city / town?

- I have always lived in the same place.
- 1 - 3
- 4 - 6
- 6 - 10
- 10+

41. What is your educational background? (Choose what education you have had or are currently studying. You can choose several options)

- Basic education
- High school / secondary school
- Vocational school or college
- Bachelor's degree
- Master's degree
- PhD or doctorate
- No education

42. Are trees and nature related to your profession?

- No
- Yes
- More or less

44. Do you own forest or other land?

- Forest property
- Property (with garden or trees) surrounding my home.
- Arable area / Field
- Arable area / Field
- I am not sure.
- I do not own a forest or land.

45. How far do you travel regularly from your home? (Every week or every month, one-way distance to the furthest place where you often travel) (Every week or every month, before the Covid-19 outbreak)

- I'm not often leaving my home.
- 0 – 5 km
- 5 – 10 km
- 10 – 20 km
- 20 – 50 km
- 50 – 100 km
- 100+ km

46. How often do you spend time in a forest or park? (Before the Covid-19 outbreak)

- I don't visit a forest or park.
- I rarely visit a forest or park.
- A few times a year
- A few times a month
- Several times a week
- Weekly
- Almost daily
- I am living in the forest.

47. How often do you spend time in a forest or park? (After the Covid-19 outbreak)

- I don't visit a forest or park.
- I rarely visit a forest or park.
- A few times a year
- A few times a month
- Several times a week
- Weekly
- Almost daily
- I am living in the forest.

48. What seasons do you prefer to go to the forest or park? (You can choose several options)

- In winter
- In spring
- In the summer
- In autumn

49. What qualities do you value most in the nature? (You can select several options)

- Beauty of nature
- Natural products
- Recreation in nature
- Financial income
- Spiritual experiences
- Biodiversity
- Other - Please describe.

50. What do you consider beautiful in nature? (You can select several options)

- Wild nature or overgrown environment
- Well maintained environment
- Greenness
- Richness of colour
- Rough and inhospitable environment
- Ecological diversity
- Peace and quietness.

- The sounds of nature
- Wilderness-like
- Other - Please explain.

51. What activities do you undertake near trees? (You can select several options)

- Walking (walks with or without a dog)
- Day hikes (hiking, trekking for a day)
- Hiking (multi-day excursions with overnight stays)
- Fitness (power walks, Nordic walks, running, biking, mountain biking, outdoor climbing, tree climbing)
- Motor Sports (motocross, ATV, snowmobiling)
- Forest management (clearing, thinning, nature management, felling, firewood)
- Gardening (weeding, planting, mowing, harvesting)
- Collecting of natural products (berry picking, mushroom picking, searching of useful plants)
- Nature sightings (observing nature in your yard, spotting birds)
- Hunting and/or fishing (hunting, fishing)
- Overnight stay (camping, hammocking)
- Art (Photography, Environmental Art, Landscape Painting)
- Relaxation (nature walking, meditation, sitting at the foot of a tree, picnic)
- Another activity - Please explain.

Are you willing to be contacted for a research interview?

Some respondents who submit their contact information will be selected for interviews. Those submitting their contact information may also opt to receive a summary of the progress and results of the study. Contact information will not be disclosed to third parties.

53. I would like to receive the Trees Near Us newsletter

- In Dutch, email-address:
- In English, email-address:
- No thanks

54. Interview

- I would like to be contacted for an interview with the Trees Near Us project. Interviews are made in English or Dutch, depending on my preference.
- No thanks

56. Do you have comments on the survey and the form?

Appendix B. Results of quantitative analysis.

We made separate NMDS analyses for human-tree relationship data, tree data, and respondent trait data (see the survey questionnaire in Appendix A). Clustering of K-medoids was applied after each NMDS. The results of NMDS and clustering are presented in Tables B1-B3. The clusters of these three analyses (human-tree relationships, trees, respondents) were then cross-tabulated and the frequency distributions were tested with χ^2 test (Tables B4-B5). We only report codes that occurred more than three times in our data and were included in the quantitative analysis. See Appendix A for all the questions presented in the survey. As an exception, we report a full list of tree taxa reported by the respondents.

Table B1.

Relationships between humans and trees.

Note. The table presents code positions in the three-dimensional ordination space (NMDS). NMDS dimensions 1 and 2 (NMDS1, NMDS2) proved to be the most essential when interpreting the NMDS solution. The codes and the respondents (n=158) were then assigned to three clusters with k-medoids clustering based on their positions along these two dimensions. After clustering, we examined how each individual code was expressed by the respondents in each three groups (columns 9-11). Based on the interpretation of NMDS and clustering results, the three clusters are called nostalgic (No), nurturing (Nu), and empowering (Em) relationship with trees. Code frequencies are reported in the last column.

ID	Code name	NMDS1	NMDS2	NMDS3	Cluster	% of No (n=35)	% of Nu (n=47)	% of Em (n=76)	n
16A	Important tree features: size	0,14	-0,13	-0,62	Em	31	30	43	58
16B	Important tree features: height	-0,02	-0,05	-0,59	Em	40	49	42	69
16C	Important tree features: blooming	-0,76	0,15	-0,15	Nu	9	34	4	22

16D	Important tree features: fruits	-0,31	0,25	-0,32	Nu	17	19	7	20
16E	Important tree features: age	0,09	-0,30	-0,53	Em	17	43	54	67
16F	Important tree features: leave colour	-0,19	0,12	-0,10	Nu	17	21	20	31
16G	Important tree features: leave shape	-0,12	0,01	0,08	Em	17	21	20	31
16H	Important tree features: autumn colour	-0,23	-0,12	0,00	Nu	11	26	14	27
16I	Important tree features: trunk shape	0,11	-0,02	-0,14	Em	23	38	46	61
16J	Important tree features: branches	-0,02	0,13	-0,24	No	31	43	38	60
16K	Important tree features: bark	0,01	-0,02	0,09	Em	23	32	36	50
16L	Important tree features: animals	-0,40	-0,25	-0,22	Nu	11	49	29	49
16M	Important tree features: location	-0,01	0,11	-0,11	Em	57	60	54	89
16N	Important tree features: scent	0,06	0,08	-0,12	Em	17	15	9	20
16O	Important tree features: sounds	-0,05	-0,37	-0,47	Em	6	15	18	23
17A	Relationship length: new	0,55	-0,50	0,12	Em	3	0	12	10
17B	Relationship length: years	0,20	-0,22	0,26	Em	31	34	66	77
17C	Relationship length: decades	-0,36	0,10	-0,25	Nu	31	43	13	41
17D	Relationship length: my life	0,07	0,69	-0,05	No	23	11	8	19
17E	Relationship length: tree life	-0,43	0,91	0,18	No	11	13	0	10
18A	Tree visits: everyday	-0,74	-0,25	-0,07	Nu	0	62	13	39
18B	Tree visits: weekly	0,01	-0,60	0,34	Em	0	23	30	34
18C	Tree visits: monthly	0,11	0,17	-0,40	No	9	2	9	11
18D	Tree visits: several times per year	0,59	0,24	0,54	No	17	2	18	21
18E	Tree visits: few times per year	0,55	-0,01	-0,15	Em	9	2	13	14
18F	Tree visits: less than once per year	0,17	0,43	0,41	No	6	0	3	4
18G	Tree visits: no more visiting	0,32	1,05	-0,23	No	60	6	7	29
20B	Emotional bond: just came to my mind	0,15	-0,32	0,23	Em	6	9	11	14
20C	Emotional bond: I see the tree often	0,03	-0,53	0,03	Em	6	19	25	30
20D	Emotional bond: part of everyday life	-0,65	-0,37	-0,07	Nu	3	53	17	39
20E	Emotional bond: brings joy	-0,12	-0,20	0,12	Em	31	79	71	102
20F	Emotional bond: admiration	0,05	-0,23	-0,11	Em	20	45	62	75
20G	Emotional bond: important memories	0,11	0,82	0,02	No	91	28	16	57
20H	Emotional bond: brings strength	0,40	-0,16	-0,09	Em	14	11	43	43
20I	Emotional bond: the tree is a friend	0,03	0,04	-0,09	Em	23	23	26	39
20J	Emotional bond: I share my worries	0,30	0,22	-0,26	No	11	2	11	13
20K	Emotional bond: represents something bigger	0,09	-0,01	0,02	Em	26	26	34	47
21G	Tree felling situation: I would do nothing	0,13	0,16	0,31	No	14	11	11	18
21A	Tree felling situation: I would understand	0,16	0,84	0,34	No	23	4	5	14
21B	Tree felling situation: I would not be involved	0,42	0,44	-0,21	No	26	4	12	20
21C	Tree felling situation: I would help felling	-0,63	0,65	0,48	Nu	0	11	3	7
21D	Tree felling situation: I would participate in resistance	0,44	-0,21	-0,25	Em	20	6	45	44
21E	Tree felling situation: I would organise resistance	-0,03	-0,34	0,20	Em	17	40	57	68
21H	Tree felling situation: I can't assess my reactions	0,13	0,16	0,31	No	14	11	11	18

21F	Tree felling situation: I would prevent felling	-0,19	-0,21	-0,07	Nu	9	45	36	51
22A	Activities: no activities reported	0,45	0,02	0,19	Em	86	9	89	102
22B	Activities: raking	-0,92	-0,18	-0,19	Nu	9	57	3	32
22C	Activities: cleaning	-0,94	-0,07	-0,26	Nu	9	53	3	30
22D	Activities: crop harvesting	-0,57	0,47	-0,27	Nu	9	17	1	12
22E	Activities: preserving	-0,98	0,07	0,27	Nu	3	53	1	27
22F	Activities: protection against pests/diseases	-1,00	0,09	0,34	Nu	0	15	0	7
22G	Activities: wood harvesting	-0,88	0,25	-0,44	Nu	0	9	0	4
22H	Activities: prevent felling	-0,47	-0,30	-0,66	Nu	0	19	7	14
23K	Relationship persistence: the favourite has been the same	0,18	0,60	-0,85	No	34	15	8	25
23U	Relationship persistence: I have many favourite trees	-0,01	-0,04	0,39	Em	51	68	76	108
24A	Selection of the tree: difficult from many candidates	-0,24	-0,18	0,43	Nu	11	17	12	21
24B	Selection of the tree: difficult to find any	0,24	0,06	0,09	Em	20	6	17	23
24C	Selection of the tree: I have only one favourite	-0,22	0,62	-0,54	No	29	26	9	29
24D	Selection of the tree: easy from many candidates	0,12	-0,13	0,22	Em	40	51	62	85
26K	Owning the tree: Yes	-0,75	0,29	0,40	Nu	26	85	8	55
26E	Owning the tree: No	0,48	-0,18	-0,06	Em	63	13	92	98

Table B2.

Trees.

Note. The table presents code positions in the three-dimensional ordination space (NMDS). NMDS dimensions 1 and 2 (NMDS1, NMDS2) proved to be the most essential when interpreting the NMDS solution. The codes and the respondents (n=158) were then assigned to three clusters with k-medoids clustering based on their positions along these two dimensions. After clustering, we examined how each individual code was expressed by the respondents in each three cluster (columns 9-11). Based on the interpretation of NMDS and clustering results, the three clusters are called everyday trees (E), garden trees (G), and charismatic trees (C). Code frequencies are reported in the last column.

**ID**	**Code name**	**NMDS1**	**NMDS2**	**NMDS3**	**Cluster**	**% of E (n=58)**	**% of G (n=50)**	**% of C (n=50)**	**n**
2A	The tree exists and lives	-0,06	-0,09	-0,03	G	76	92	74	127
2B	The tree exists, but is dead	0,68	-0,32	0,27	C	2	0	8	5
2C	There is no more that tree	0,14	0,53	0,21	E (C)	10	2	8	11
2D	I don't know whether the tree still exists	0,43	-0,05	-0,63	C (E)	5	2	6	7

3A	Tree location: own yard	-0,60	0,05	0,01	G	24	38	8	37
3B	Tree location: close my home	-0,03	-0,01	0,51	E	33	28	22	44
3C	Tree location: close my former home	0,14	-0,01	-0,32	C	9	8	16	17
3D	Tree location: yard of my friend/relatives	-0,28	-0,21	-0,83	G (E)	7	8	2	9
3E	Tree location: at or along the way to my workplace/school	0,41	0,12	-0,55	C (E)	5	2	6	7
3F	Tree location: at town	0,47	0,32	-0,05	C (E)	12	4	16	17
3G	Tree location: abroad	0,44	-0,38	-0,13	C	3	4	8	8
3H	Tree location: at my current hometown	-0,17	0,06	0,19	E (G)	67	70	48	98
4A	Tree surroundings: street	0,17	0,24	0,24	E (C)	16	8	16	21
4B	Tree surroundings: park	0,07	0,02	0,79	E	19	14	16	26
4C	Tree surroundings: yard	-0,53	0,03	-0,22	G	47	62	6	61
4D	Tree surroundings: forest edge	0,73	0,03	-0,52	C	7	0	24	16
4E	Tree surroundings: forest	0,55	-0,22	-0,23	C	0	8	18	13
4F	Tree surroundings: hill	0,64	-0,46	-0,41	C	3	0	12	8
4G	Tree surroundings: shore	0,47	0,04	0,00	C	3	2	12	9
4H	Tree surroundings: agricultural landscape[1]	0,27	-0,46	0,07	C	2	6	10	9
5A	Tree type: deciduous	0,03	0,03	-0,11	E	91	82	94	141
5B	Tree type: coniferous	-0,37	-0,41	0,91	G	9	16	4	15
5C	Tree type: native	0,14	-0,06	-0,11	C	74	60	90	118
5D	Tree type: exotic	-0,58	0,01	0,59	G	17	28	4	26
5E	Tree type: fruit	-0,56	0,04	-0,41	G	5	8	0	7
5F	Tree type: nut	-0,03	0,13	0,02	E	2	6	6	7
6A	Tree taxon: I don't know	0,04	-0,70	-0,32	G	3	8	0	6
6B	Tree taxon: oak	0,38	-0,35	-0,10	C	9	10	32	26
6C	Tree taxon: birch	-0,47	0,80	-0,44	E	22	2	0	14
6D	Tree taxon: aspen	-0,14	0,35	-0,09	E	10	6	0	9
6E	Tree taxon: beech	0,52	-0,16	-0,15	C	2	10	30	21
6F	Tree taxon: plane	0,68	0,37	0,46	C	2	0	6	4
6G	Tree taxon: weeping willow	0,17	-0,21	-0,45	C	2	2	4	4
6H	Tree taxon: willow	-0,10	-0,07	0,05	G	3	6	2	6
6I	Tree taxon: pine	-0,32	-0,84	1,06	G	2	4	2	4
6J	Tree taxon: maple	-0,22	-0,06	-0,22	G	2	4	2	4
6K	Tree taxon: maidenhair tree	-0,87	-0,25	0,43	G	2	6	0	4
6L	Tree taxon: horse chestnut	0,51	0,22	0,56	C	3	0	6	5
7A	Tree origin: natural	0,10	-0,56	-0,43	G	7	6	8	11
7B	Tree origin: I planted	-0,85	-0,48	-0,65	G	3	30	0	17
7C	Tree origin: planted by someone I don't know	-0,24	0,21	0,61	E	59	34	14	58
7D	Tree origin: planted by someone close to me	-0,34	0,15	-0,20	E (G)	14	14	4	17
7E	Tree origin: I don't know the origin	0,58	-0,08	-0,31	C	17	16	74	55
8A	Tree stands alone	-0,03	-0,07	0,07	G	72	80	74	119
8B	Tree belongs to a group of trees	0,06	0,11	-0,24	E	28	20	26	39
9A	Tree age: 0-20 years	-0,88	-0,66	-0,66	G	0	24	0	12

9B	Tree age: 20-50 years	-0,37	0,33	-0,01	E	45	30	2	42
9C	Tree age: 50-100 years	-0,07	0,25	0,04	E	38	20	16	40
9D	Tree age: more than 100 years	0,65	-0,32	0,29	C	3	8	64	38
10A	Tree height: 0-2 meters	-0,98	-0,63	-0,99	G	0	10	0	5
10B	Tree height: 1.5-3 meters	-0,39	-0,86	-0,33	G	2	14	2	9
10C	Tree height: 3-9 meters	-0,28	0,13	-0,28	E (G)	48	42	12	55
10D	Tree height: 9-20 meters	0,06	0,10	0,21	E (C)	38	26	36	53
10E	Tree height: 20-30 meters	0,52	-0,29	0,29	C	7	8	36	26
10F	Tree height: more than 30 meters	0,59	0,24	0,36	C	5	0	14	10
11A	Tree circumference: 3-10 cm	-0,95	-0,77	-1,04	G	0	8	0	4
11B	Tree circumference: 20-40 cm	-0,87	-0,47	-0,50	G	2	16	0	9
11C	Tree circumference: 40-80 cm	-0,57	-0,14	-0,54	G (E)	10	16	0	14
11D	Tree circumference: 80-150 cm	-0,47	0,47	-0,03	E	40	14	4	32
11E	Tree circumference: 150-180 cm	0,03	0,01	-0,26	E (G)	19	22	12	28
11F	Tree circumference: more than 180 cm	0,48	-0,12	0,35	C	28	22	84	69
12A	Autumn colour: green as always	-0,38	-0,34	0,47	G	7	16	6	15
12B	Autumn colour: pale	-0,20	0,02	-0,35	G	12	10	10	17
12C	Autumn colour: yellow	-0,32	0,35	0,04	E	45	22	10	42
12D	Autumn colour: red	0,32	-0,32	0,13	C	7	16	30	27
12E	Autumn colour: multicolour	0,30	0,06	-0,24	C	14	18	26	30
12F	Autumn colour: leaves are dropped green	-0,27	-0,45	1,14	G	2	4	2	4
12G	Autumn colour: I don't know	0,19	-0,30	-0,21	C	12	14	14	21
13A	Tree scent: bark	0,15	0,45	0,14	E (C)	19	4	16	21
13B	Tree scent: leaves	0,27	0,76	0,12	E	26	0	14	22
13C	Tree scent: flowers	-0,50	0,30	-0,12	E	9	10	2	11
13D	Tree scent: twigs	0,06	0,59	0,24	E	12	4	6	12
13E	Tree scent: resin	-0,03	0,27	0,43	E	12	6	4	12
13F	Tree scent: decay	0,43	0,23	-0,05	C	17	6	24	25
13G	Tree scent: unimportant[2]	-0,06	-0,27	-0,04	G	41	72	54	87
14A	Sound sources: trunk	0,19	0,81	0,46	E	10	2	4	9
14B	Sound sources: leaves	0,08	0,55	-0,14	E	86	12	50	81
14C	Sound sources: branches	0,19	0,65	0,08	E	38	2	28	37
14D	Sound sources: animals	-0,08	0,52	0,07	E	24	4	10	21
14E	Sound sources: sound unimportant[2]	-0,15	-0,83	0,00	G	2	86	38	63
15A	Trunk shape: straight	-0,32	-0,02	0,17	G (E)	34	40	10	45
15B	Trunk shape: branched	0,09	0,19	-0,28	E	22	22	28	38
15C	Trunk shape: burls	0,25	0,10	-0,15	C	24	20	36	42
15D	Trunk shape: regular	-0,27	0,16	0,13	E	19	14	10	23
15E	Trunk shape: curved	0,22	-0,01	-0,04	C	26	24	42	48
15F	Trunk shape: thick	0,34	0,00	0,24	C	24	22	56	53
15G	Trunk shape: slim	-0,52	-0,21	-0,80	G	3	8	0	6
15H	Trunk shape: many twigs	0,33	0,10	0,03	C	21	16	46	43
15I	Trunk shape: few twigs	-0,30	-0,14	-0,39	G	3	10	0	7

[1] Subsequently, the code was created based on Question 4 in the questionnaire, answer to the open field: “other – what”. (Appendix A).
[2]The code covers all who did not report any scents/sounds, not only those who answered, “I don’t know” (Appendix A).

Table B3.

Respondents.

Note. The table presents code positions in the five-dimensional ordination space (NMDS). NMDS dimensions 1 and 2 (NMDS1, NMDS2) proved to be the most essential when interpreting the NMDS solution. The codes and the respondents (n=158) were then assigned into three clusters with k-medoids clustering based on their positions along these two dimensions. After clustering, we examined how each individual code was expressed by the respondents in each three cluster (columns 9-11). Based on the interpretation of NMDS and clustering results, the three clusters are called older city dwellers (Oc), younger city dwellers (Yc), and older country dwellers (Co). Code frequencies are reported in the last column.

ID	Code name	NMDS1	NMDS2	NMDS3	NMDS4	NMDS5	Cluster	% of Oc (n=58)	% of Yc (n=43)	% of Co (n=57)	n
27A	Age: 0-29	0,71	0,13	0,25	-0,12	-0,68	Yc	14	51	5	33
27B	Age: 30-49	0,30	0,23	-0,09	-0,05	-0,18	Yc	16	28	21	33
27C	Age: 50-69	-0,41	-0,30	-0,11	0,11	0,33	Oc	67	16	65	83
27D	Age: 70 or more	-0,67	0,56	0,87	-0,14	0,04	Co	3	2	9	8
28A	Gender identity: man	-0,29	-0,48	0,62	-0,70	-0,02	Oc	50	14	28	51
28B	Gender identity: woman	0,08	0,14	-0,28	0,37	0,01	Yc	50	81	70	104
31A	Living environment: city	0,41	-0,41	-0,17	-0,44	0,20	Oc	40	40	5	43
31B	Living environment: suburb	-0,04	-0,05	-0,21	0,00	0,03	Oc	9	12	11	16
31C	Living environment: town	0,31	-0,23	0,22	0,34	-0,04	Oc	33	33	14	41
31D	Living environment: village	-0,51	0,27	-0,06	0,04	-0,17	Co	12	16	46	40
31E	Living environment: countryside	-0,91	0,49	0,57	0,33	-0,21	Co	5	0	21	15
32A	I still live where I was born	-0,13	-0,40	0,31	0,34	-0,21	Oc	26	14	18	31
33A	Identity: urban	0,35	-0,72	-0,09	-0,11	0,10	Oc	36	23	4	33
33B	Identity: countryman	-0,52	0,25	0,46	0,27	-0,23	Co	22	16	58	53
33C	Identity: both urban and countryman	0,18	-0,08	-0,41	0,00	0,17	Oc	29	35	25	46
33D	Identity: neither urban nor countryman	0,17	0,31	0,01	-0,33	0,03	Yc	10	26	14	25
34A	Housing: own apartment (block)	0,46	-0,46	-0,08	-0,15	-0,03	Oc	14	9	4	14
34B	Housing: hired apartment (block)	0,96	-0,09	0,05	-0,02	0,18	Yc	7	44	0	23
34C	Housing: own apartment (rowhouse)	-0,26	-0,22	-0,10	0,23	0,01	Oc	60	16	42	66
34D	Housing: hired apartment (rowhouse)	0,11	0,16	0,74	-0,01	0,13	Yc	5	9	7	11

34E	Housing: hired house	-0,80	0,36	0,11	-0,34	-0,07	Co	5	0	37	24
34F	Housing: own house or farm	-0,61	0,27	0,23	-0,29	-0,12	Co	3	5	9	9
34G	Housing: dormitory	0,78	0,25	-0,16	-0,06	-0,98	Yc	3	12	0	7
35A	Moving: never	0,21	-0,27	0,80	0,69	-0,47	Oc	7	7	5	10
35B	Moving: 1-3 times	0,15	-0,30	-0,25	0,27	-0,30	Oc	48	44	21	59
35C	Moving: 4-6 times	-0,22	0,23	0,16	-0,41	0,12	Co	21	26	39	45
35D	Moving: 7-10 times	-0,12	0,24	0,33	-0,03	0,28	Co	9	23	28	31
35E	Moving: more than 10 times	-0,22	-0,51	-0,67	-0,21	0,61	Oc	16	0	5	12
36A	Education: basic	0,56	-0,03	0,22	0,41	-0,40	Yc	10	21	7	19
36B	Education: high school	0,17	-0,43	0,27	0,32	-0,30	Oc	26	21	12	31
36C	Education: college	-0,34	-0,66	0,12	0,30	0,44	Oc	31	5	12	27
36D	Education: bachelor	0,36	0,17	0,26	0,24	-0,08	Yc	19	51	28	49
36E	Education: master	-0,07	0,07	-0,31	-0,23	-0,18	Co	34	44	51	68
36F	Education: PhD	-0,25	0,24	0,13	-0,84	0,16	Co	7	2	9	10
37A	Working as a nature professional: yes	-0,23	-0,31	-0,08	0,29	0,25	Oc	59	26	46	71
37B	Working as a nature professional: kind of	0,32	0,34	0,43	0,07	-0,13	Yc	9	37	21	33
38A	Property: house	-0,65	0,20	-0,25	-0,02	-0,02	Co	33	9	88	73
38B	Property: cottage	0,47	0,34	-0,48	-0,82	0,66	Yc	3	7	0	5
38C	Property: no land property	0,54	-0,33	0,38	0,11	-0,03	Oc	59	77	9	72
39A	Regular travelling: 0-10 km from home	0,08	-0,06	-0,16	0,45	-0,23	Oc	21	26	21	35
39B	Regular travelling: 10-50 km from home	-0,14	0,02	0,30	-0,10	0,48	Co	40	30	47	63
39C	Regular travelling: more than 50 km from home	0,00	-0,10	-0,18	-0,15	-0,38	Oc	36	40	30	55
39D	Regular travelling: not any	-0,13	-0,32	-0,30	-0,14	-0,14	Oc	3	2	2	4
40A	Visits in forest/park: I live in forest	-0,25	0,88	-0,90	-0,79	0,19	Co	0	2	5	4
40B	Visits in forest/park: daily or near so	0,15	0,31	0,18	0,10	0,58	Yc	26	63	44	67
40C	Visits in forest/park: weekly or near so	-0,14	-0,47	-0,15	-0,16	-0,58	Oc	62	30	35	69
40D	Visits in forest/park: I rarely visit	-0,32	0,07	0,35	0,53	0,06	Co	10	5	16	17
40E	Visits in forest/park: more after COVID-19 outbreak	0,32	-0,33	-0,41	0,13	-0,62	Oc	17	26	5	24
40F	Visits in forest/park: less after COVID-19 outbreak	0,23	0,14	0,48	0,37	-0,01	Yc	12	30	5	23
41A	Favourite season for forest/park visits: winter	-0,03	-0,19	-0,16	-0,08	0,21	Oc	69	49	49	89
41B	Favourite season for forest/park visits: spring	0,03	-0,08	-0,03	0,03	0,08	Oc	91	91	84	140
41C	Favourite season for forest/park visits: summer	-0,01	-0,13	-0,10	0,02	0,05	Oc	79	70	63	112
41D	Favourite season for forest/park visits: autumn	-0,05	-0,09	-0,06	0,08	0,00	Oc	90	84	82	135
42A	Valuable qualities of nature: beauty	0,03	0,00	0,06	0,05	-0,02	Oc	86	100	91	145
42B	Valuable qualities of nature: natural products	0,18	0,25	-0,28	-0,13	0,09	Yc	17	28	21	34
42C	Valuable qualities of nature: recreation	0,27	0,00	-0,28	-0,18	0,11	Yc	34	51	33	61
42D	Valuable qualities of nature: spiritual experiences	-0,04	0,11	-0,25	-0,21	-0,02	Co	38	44	47	68

42E	Valuable qualities of nature: biodiversity	0,01	0,19	0,04	-0,19	-0,06	Co	57	81	79	113
42F	Valuable qualities of nature: peace and silence[1]	0,17	0,16	0,09	0,27	0,09	Yc	3	14	9	13
42G	Valuable qualities of nature: space and freedom[1]	0,63	0,04	0,20	-0,07	0,29	Yc	2	12	0	6
42H	Valuable qualities of nature: nature as it is	-0,01	0,06	-0,32	0,12	0,18	Co	9	5	5	10
43A	Beautiful in nature: wild/overgrown nature	0,04	0,02	-0,02	0,06	-0,02	Oc	83	93	82	135
43B	Beautiful in nature: maintained nature	-0,01	-0,11	0,17	0,34	-0,04	Oc	9	16	7	16
43C	Beautiful in nature: greenness	0,21	0,16	-0,06	0,07	0,22	Yc	38	67	47	78
43D	Beautiful in nature: colours	0,02	0,24	0,01	0,18	-0,05	Co	34	65	65	85
43E	Beautiful in nature: roughness	0,12	0,07	0,12	-0,08	-0,07	Yc	60	77	61	103
43F	Beautiful in nature: biodiversity	0,00	0,12	0,04	0,01	-0,08	Co	66	84	84	122
43G	Beautiful in nature: peace	0,08	0,06	-0,01	0,03	0,13	Yc	66	79	72	113
43H	Beautiful in nature: sounds	0,08	0,08	0,07	0,03	-0,02	Yc	74	95	88	134
43I	Beautiful in nature: wilderness-like nature	0,16	0,19	0,13	0,00	-0,11	Yc	48	81	61	98
44A	Activities near trees: walking	-0,01	0,03	-0,04	0,02	-0,06	Co	84	95	93	143
44B	Activities near trees: day hike	0,14	0,13	-0,05	-0,26	0,10	Yc	43	51	40	70
44C	Activities near trees: hiking	0,16	0,31	-0,12	-0,46	0,11	Yc	17	33	21	36
44D	Activities near trees: fitness	0,34	-0,11	0,02	-0,22	0,08	Yc	34	47	21	52
44E	Activities near trees: forestry	-0,28	0,31	-0,11	-0,45	0,34	Co	12	12	23	25
44F	Activities near trees: gardening	-0,17	0,20	-0,26	-0,02	0,12	Co	40	44	67	80
44G	Activities near trees: collecting	-0,08	0,41	-0,41	-0,41	-0,40	Co	17	28	35	42
44H	Activities near trees: observing	-0,09	0,25	0,07	0,00	-0,07	Co	47	77	93	113
44I	Activities near trees: camping	0,17	0,27	-0,20	-0,36	0,16	Yc	22	42	30	48
44J	Activities near trees: art	0,03	0,20	0,01	-0,02	-0,13	Co	26	51	51	66

[1] The code has been created afterward based on question 42 in the questionnaire, answering the open field: 'other - what'. (Appendix A).

Table B4.

Cross-tabulation of the three clusters of human-tree relationship and the three clusters of trees.

Note. Differences between observed and expected frequencies were tested with χ^2 test. The table shows the observed frequencies and deviations from the expected frequencies in percentages. To locate significant differences better – and because we were interested in positive associations between the clusters - we only tested whether cluster frequencies with over 30 % positive deviation from the expected frequency within each human-tree relationship cluster differed from the expected frequencies, when the other two clusters collapsed. This resulted in five χ^2 tests and we corrected our level of significant p-values accordingly to 0.01. Values between 0.01 and 0.05 were interpreted as nearly significant.

	**Nostalgic relationship**	**Nurturing relationship**	**Empowering relationship**	Row sum
**Everyday trees**	18 (+40 %)	18 (+4 %)	22 (-21 %)	58

**Garden trees**	7 (-37 %)	24 (+61 %)	19 (-21 %)	50
**Charismatic trees**	10 (-10 %)	5 (-66 %)	35 (+46 %)	50
Column sum	35	47	76	158
Test	Everyday trees vs. other two clusters collapsed χ^2 (1) =3.3, p=0.071	Garden trees vs. other two clusters collapsed χ^2 (1) =8.2, p=0.004	Charismatic trees vs. other two clusters collapsed χ^2 (1) =7.3, p=0.007	

Table B5.

Cross-tabulation of the three clusters of human-tree relationship and the three clusters of respondent traits.

Note. Differences between observed and expected frequencies were tested with χ^2 test. The table shows the observed frequencies and deviations from the expected frequencies in percentages. To locate significant differences better, we skipped the universal test and conducted three separate tests within the human-tree relationships clusters. No post hoc tests were conducted after this. As we had in total six tests in our study, we corrected our significance level to 0.008. Values between 0.008 and 0.05 were interpreted as nearly significant.

	**Nostalgic relationship**	**Nurturing relationship**	**Empowering relationship**	Row sum
**Older city dwellers**	13 (+1 %)	18 (+4)	27 (-3 %)	58
**Younger city dwellers**	15 (+57 %)	3 (-77 %)	25 (+21 %)	43
**Older country dwellers**	7 (-45 %)	26 (+53 %)	24 (-12 %)	57
Column sum	35	47	76	158
Test	Younger city dwellers vs. other two clusters collapsed χ^2 (1) =4.3, p=0.038	Older country dwellers vs. other two cluster collapsed χ^2 (1) =7.5, p=0.006	-	

Table B6.

List of tree taxa.

Note. The table also includes those taxa that were reported in the open field after the provided tree list (Appendix A).

**Taxon**	**English name**	**Dutch name**	**n**
Quercus robur	English Oak	Zomereik / Eik	27
Fagus sylvatica	European Beech	Beuk	23
Betula pendula / pubescens	Silver Birch / Downy Birch	Ruwe berk / Zachte berk	16
Populus tremula	Aspen	Ratelpopulier / Populier	9
Salix sp.	Willow	Wilg	7
Aesculus hippocastanum	Horse Chestnut	Paardenkastanje	5
Pinus sylvestris	Scots Pine	Grove den	5
Acer platanoides	Norway Maple	Noorse Esdoorn	4
Ginko biloba	Maidenhair Tree/Ginko	Ginko	4
Platanus acerifolia	London Plane Tree	Gewone Plataan	4

Salix sepulcralis	White Weeping Willow	Treurwilg	4
Fraxinus excelsior	European Ash	Gewone Es	3
Juglans regia	Common Walnut	Walnoot	3
Robinia pseudoacacia	Black Locust	Acasia	3
Tilia cordata/ Tilia × vulgaris	Linden	Linde	3
Ulmus sp.	Elm	Lep	3
Castanea sativa	Sweet Chestnut	Tamme kastanje	2
Larix sp.	Larch	Lariks	2
Picea abies	Norway Spruce	Fijnspar / Spar	2
Prunus domestica	Plum	Pruimenboom	2
Prunus sp.	Cherry Tree	Kersenboom	2
Pterocarya sp.	Wingnut	Vleugelnoot	2
Acer platanoides 'Royal Red'	Royal Red Norway Maple	Purperen Noorse esdoorn	1
Ceiba sp.	Ceiba	Ceiba	1
Ceratonia siliqua	Carob Tree	Johannesbroodboom	1
Colophospermum mopane	Mopane Tree	Mopaneboom	1
Cupressaceae	Cypress Family	Cipresfamilie	1
Eriobotrya japonica	Loquat	Loquat	1
Koelreuteria sp.	Goldenrain Tree	Lampionboom	1
Malus domestica	Apple Tree	Appelboom	1
Mespilus germanica	Medlar	Mispelboom	1
Metasequoia glyptostroboides	Dawn Redwood	Watercipres	1
Morus sp.	Mulberry	Moerbeiboom	1
Olea europaea	Olive Tree	Olijfboom	1
Prunus serrulata	Japanese Cherry Blossom	Japanse kers	1
Pyrus communis	Pear Tree	Perenboom	1
Salix caprea	Goat Willow	Boswilg	1
Sassasfras sp.	Sassafras	Sassafras	1
Sequioa sp.	Sequoia	Sequoia	1
Sorbus aucuparia	Rowan	Lijsterbes	1
Thuja sp.	Arborvitae / Thuja	Levensboom / Thuja	1
---	Maypole	Meiboom (Limburgse benaming)	1
